# Consumption of sugar sweetened beverages, artificially sweetened beverages, and fruit juice and incidence of type 2 diabetes: systematic review, meta-analysis, and estimation of population attributable fraction

**DOI:** 10.1136/bmj.h3576

**Published:** 2015-07-21

**Authors:** Fumiaki Imamura, Laura O’Connor, Zheng Ye, Jaakko Mursu, Yasuaki Hayashino, Shilpa N Bhupathiraju, Nita G Forouhi

**Affiliations:** 1 Medical Research Council Epidemiology Unit, University of Cambridge School of Clinical Medicine, Institute of Metabolic Science, Cambridge, CB2 0QQ, UK; 2 Institute of Public Health and Clinical Nutrition, University of Eastern Finland, Kuopio, Finland; 3 Department of Endocrinology, Tenri Hospital, Tenri City, Nara, Japan; 4 Department of Healthcare Epidemiology, Graduate School of Medicine and Public Health, Kyoto University, Sakyo-ku, Kyoto, Japan; 5 Department of Nutrition, Harvard T H Chan School of Public Health, Boston, MA, USA

## Abstract

**Objectives** To examine the prospective associations between consumption of sugar sweetened beverages, artificially sweetened beverages, and fruit juice with type 2 diabetes before and after adjustment for adiposity, and to estimate the population attributable fraction for type 2 diabetes from consumption of sugar sweetened beverages in the United States and United Kingdom.

**Design** Systematic review and meta-analysis.

**Data sources and eligibility** PubMed, Embase, Ovid, and Web of Knowledge for prospective studies of adults without diabetes, published until February 2014. The population attributable fraction was estimated in national surveys in the USA, 2009-10 (n=4729 representing 189.1 million adults without diabetes) and the UK, 2008-12 (n=1932 representing 44.7 million).

**Synthesis methods** Random effects meta-analysis and survey analysis for population attributable fraction associated with consumption of sugar sweetened beverages.

**Results** Prespecified information was extracted from 17 cohorts (38 253 cases/10 126 754 person years). Higher consumption of sugar sweetened beverages was associated with a greater incidence of type 2 diabetes, by 18% per one serving/day (95% confidence interval 9% to 28%, I^2^ for heterogeneity=89%) and 13% (6% to 21%, I^2^=79%) before and after adjustment for adiposity; for artificially sweetened beverages, 25% (18% to 33%, I^2^=70%) and 8% (2% to 15%, I^2^=64%); and for fruit juice, 5% (−1% to 11%, I^2^=58%) and 7% (1% to 14%, I^2^=51%). Potential sources of heterogeneity or bias were not evident for sugar sweetened beverages. For artificially sweetened beverages, publication bias and residual confounding were indicated. For fruit juice the finding was non-significant in studies ascertaining type 2 diabetes objectively (P for heterogeneity=0.008). Under specified assumptions for population attributable fraction, of 20.9 million events of type 2 diabetes predicted to occur over 10 years in the USA (absolute event rate 11.0%), 1.8 million would be attributable to consumption of sugar sweetened beverages (population attributable fraction 8.7%, 95% confidence interval 3.9% to 12.9%); and of 2.6 million events in the UK (absolute event rate 5.8%), 79 000 would be attributable to consumption of sugar sweetened beverages (population attributable fraction 3.6%, 1.7% to 5.6%).

**Conclusions** Habitual consumption of sugar sweetened beverages was associated with a greater incidence of type 2 diabetes, independently of adiposity. Although artificially sweetened beverages and fruit juice also showd positive associations with incidence of type 2 diabetes, the findings were likely to involve bias. None the less, both artificially sweetened beverages and fruit juice were unlikely to be healthy alternatives to sugar sweetened beverages for the prevention of type 2 diabetes. Under assumption of causality, consumption of sugar sweetened beverages over years may be related to a substantial number of cases of new onset diabetes.

## Introduction

The health effects of sugar sweetened beverages, artificially sweetened beverages, and fruit juice have received considerable attention from scientific and public communities. The consumption of sugar sweetened beverages is likely to contribute to an increase in obesity and the development of type 2 diabetes.^1^ ^2^ ^3^ ^4^ ^5^ Artificially sweetened beverages and fruit juice are candidate alternatives to sugar sweetened beverages, but their prospective associations with type 2 diabetes have not yet been well established because only a few studies have examined the associations, of which potential bias has been debated.^5^ ^6^ ^7^ ^8^ ^9^

Each of these beverage types has been investigated and reviewed for prospective associations with incident type 2 diabetes.^4^ ^5^ ^6^ ^7^ ^8^ ^9^ A few quantitative reviews were available, but one aggregated studies that did and did not adjust for obesity status^4^ ^9^ and the other separated such studies ad hoc only for those of sugar sweetened beverages and not for those of artificially sweetened beveraor fruit juice.^5^ It is crucial to better characterise the influence of adiposity because obesity can directly cause type 2 diabetes and thus mediate an association between consumption of sugar sweetened beverages and type 2 diabetes^4^ ^5^; and because obesity can be a confounder by altering dietary habits and confounding an association between beverage consumption and incident type 2 diabetes.^8^ ^10^ Previous studies indeed reported that obese individuals tend to consume more sugar sweetened and artificially sweetened beverages and less fruit juice than leaner individuals.^6^ ^10^ ^11^ Moreover, despite the growing interest in a policy intervention to reduce the consumption of sugar sweetened beverages at a population level,^12^ ^13^ ^14^ no study has translated a prospective association between consumption of sugar sweetened beverages and type 2 diabetes into a measure of its population level impact, including population attributable fraction, in a contemporary population.

We therefore conducted a systematic review and meta-analysis of prospective studies to test whether or not habitual consumption of sugar sweetened beverages, artificially sweetened beverages, or fruit juice would be associated with the incidence of type 2 diabetes. We specifically aimed to meta-analyse the associations with and without adjustment for adiposity, because the association may be both mediated and confounded by this factor. To provide policy relevant measures, we then used the result of the meta-analysis for sugar sweetened beverages to estimate the population attributable fraction for the 10 year risk of developing type 2 diabetes due to consumption of sugar sweetened beverages in contemporary populations of the United States and United Kingdom, where approximately half of each population in recent years consumed sugar sweetened beverages.^1^ ^10^

## Methods

### Patient involvement

There was no patient involvement in this study.

### Study searches and selection

Following the PRISMA guidelines^15^ and the protocol (not registered, available on request), we identified relevant studies through hand searches and systematic searches of four databases on 31 May 2013 (updated on 10 February 2014): PubMed, Embase, Ovid, and Web of Knowledge. Search terms included those related to types of beverages, diabetes, and prospective study design (see supplementary information for details). Time and language of publications were not restricted. After the removal of duplicates, one author (FI) screened the articles on the basis of the titles and abstracts and three authors (FI, LO’C, and ZY) independently reviewed them in duplicate. We considered studies to be eligible for inclusion if they were of a prospective design, assessed the consumption of beverages and incident type 2 diabetes, and recruited adults free of diabetes and aged 18 years or older. We also considered a follow-up of at least two years on average because incidence of diabetes could alter approximately two years after modification of lifestyle.^16^ ^17^

### Data extraction and quality assessment

We extracted information in a standardised manner in duplicate, including baseline personal information such as body mass index, and duration of follow-up, exclusion criteria, sample size, loss to follow-up, assessments of beverage consumption and incident type 2 diabetes, types of beverage consumed, measures of prospective associations with 95% confidence intervals, covariates evaluated, and sources of funding. We extracted measures of associations that were the most adjusted for sociodemographic and lifestyle factors, with and without further adjustment for adiposity measures. Although adjustment for total energy intake is important to assess,^4^ ^6^ in this meta-analysis we used estimates adjusted for total energy whenever possible for parsimony and potentials for energy adjustment to reduce confounding and measurement errors.^18^ We extracted estimates stratified by age, sex, and adiposity measures, if reported, to use in meta-regression to assess heterogeneity. Additional information on study design and quality was also obtained from identified articles as well as from relevant articles of identified cohorts.

We contacted authors of identified articles to request additional information if the article did not report two types of estimates before and after adjustment for adiposity, based on either categorical or continuous analysis for sugar sweetened beverages, artificially sweetened beverages, and fruit juice separately. When we contacted authors we requested estimates before and after adjustment for adiposity based on both continuous and categorical variables of each beverage consumed, and we requested estimates based on longer follow-up if available. In addition we contacted authors of cohorts that did not meet eligibility criteria but could be eligible on the provision of additional information (see supplementary table S1).

We examined risks of bias in concordance with the Cochrane tools, including a Cochrane risk of bias assessment tool for non-randomised studies of interventions.^19^ ^20^ ^21^ Seven domains were assessed: confounding, selection, exposure measurement, misclassification over time, missing data, outcome measurement, and selective reporting. Bias specific to this meta-analysis included the likelihood of misclassifying sugar sweetened fruit drink as fruit juice (for example, fruit punch). Sources of bias were evaluated by using meta-regression for each as a potential source of heterogeneity, meta-analysis excluding studies with a certain type of bias, or meta-analysis incorporating quantitative measures of bias (see supplementary information). Overall quality of evidence was assessed based on study quality, results from sensitivity analysis, and principles of the grades of recommendation, assessment, development, and evaluation (GRADE).^22^ One author (FI) first summarised the results of bias assessment and quality of overall evidence and these results were discussed among the other authors (FI, LOC, YZ, and NGF) for consensus.

### Meta-analysis

We used Stata 13.1 for analyses (α two sided P=0.05, unless indicated). Statistical details are described in the supplementary information. Each of sugar sweetened beverages, artificially sweetened beverages, and fruit juice was considered as the main exposure. We defined sugar sweetened beverages as any sweetened beverages, including sugar sweetened fruit juice, not presented as diet or non-caloric beverages. Artificially sweetened beverages included low caloric soft drinks as reported in each study. Fruit juice was defined as 100% fruit juice, or fruit juice assessed separately from fruit drinks. We standardised measures of associations to relative risk per one serving/day of beverage consumption, after we confirmed that this unit was the most frequently used in studies. Because volume per serving was specific to a population, ranging from 237 mL (one cup) to 355 mL (12 oz) (median across publications=250 mL/day), we repeated meta-analysis to estimate the relative risk for each 250 mL/day. We converted odds ratios, if reported, to relative risks.^23^ If a study reported categorical estimates only, they were combined to obtain a single dose-response estimate.^24^ If only stratified estimates were reported, we merged them by fixed effects meta-analysis to derive a cohort specific estimate, assuming consistency of associations within a cohort.

We performed random effects meta-analysis as prespecified, assuming that biological effects of beverages in different populations would vary randomly at least by processing and composition of beverages. The heterogeneity of associations was expressed by I^2^.^25^ For each of the beverages of interest, we estimated relative risks before and after adjustment for adiposity measures. To assess a magnitude of overall confounding, we additionally estimated crude relative risks without any adjustment. Non-linear associations were additionally evaluated by cubic spline meta-analysis for which we used available categorical estimates.^24^

In observational studies, within person variability of exposure can cause bias.^26^ ^27^ ^28^ ^29^ ^30^ As performed previously,^26^ ^27^ ^28^ ^29^ ^30^ ^31^ ^32^ we compiled within person variation of beverage consumption in each study and adjusted for them to estimates of each study. Uncertainty in self reported diagnosis of type 2 diabetes was also calibrated for estimates from studies without objective information on incidence of type 2 diabetes.^33^ To compute relative risks adjusted for within person dietary variation and uncertainty of type 2 diabetes ascertainment, we pooled the estimates after study specific calibration.

Meta-regression was used to assess if heterogeneity of associations across studies depended on population demographics, study characteristics, and indicators of errors or bias. Publication status (peer reviewed or not), selective reporting (yes or no), and mutual adjustment for three beverage types were evaluated after we identified studies with those characteristics. Stratified meta-analysis was performed by each variable that predicted heterogeneity (P<0.01) and by prespecified variables: age, sex, body mass index, and study location. In exploratory analysis using multiple variables of study specific factors, we reassessed I^2^ as a magnitude of unexplained heterogeneity.

Publication bias was assessed by Egger’s test, with a contour enhanced funnel plot, and ‘trim and fill’ analysis.^34^ If publication bias was indicated, we adjusted summary estimates for the bias.^34^ Robustness of summary findings was examined by sensitivity analyses: influence analysis,^32^ fixed effects meta-analysis, analysis using millilitres per day as a unit, analysis without studies with a high overall risk of bias, and analysis incorporating measures of uncertainty in adjustment for within person dietary variations and diagnosis of type 2 diabetes.^35^

Adiposity is likely to confound an association of beverage consumption with type 2 diabetes, particularly in research on artificially sweetened beverages.^6^ ^8^ ^10^ ^11^ Because of imperfect measurement of adiposity in an epidemiological study,^36^ adjustment for adiposity was likely to be insufficient, as discussed previously.^3^ ^6^ ^32^ ^37^ ^38^ ^39^ ^40^ ^41^ Thus, to assess if such residual confounding would be substantial, we performed simulation analysis to examine the influence of the bias.^42^

### Type 2 diabetes risk attributable to sugar sweetened beverages in USA and UK

We estimated the risks of type 2 diabetes attributable to consumption of sugar sweetened beverages over 10 years in the USA and UK.^43^ ^44^ These countries contributed to the meta-analysis to the largest extent and provided publically available data on diets and risk factors for type 2 diabetes: the US national health and nutrition examination survey, 2009-10^45^ and the UK national diet and nutrition survey, 2008-12.^46^ The recent cycle was selected for greater generalisability to recent populations. Selecting adults aged 20 years or more and without prevalent diabetes, we analysed 4729 US adults and 1932 UK adults. Accounting for sampling weight, 189.1 million US adults and 44.7 million UK adults were represented.

We estimated the population attributable fraction by applying a Cochrane Collaboration algorithm to survey data.^43^ ^44^ We first estimated habitual consumption of sugar sweetened beverages based on 24 hour recalls in the USA and four day food records in the UK. Then we estimated the 10 year risk of type 2 diabetes based on a risk prediction algorithm developed and validated in each country.^47^ ^48^ The predicted risk for each individual was considered as an “assumed control risk”^44^ if the current consumption of sugar sweetened beverages would remain constant. Then we calculated an alternative risk if the consumption of sugar sweetened beverages would become zero, calculating assumed control risk×(1/relative risk per serving/day)×observed sugar sweetened beverages servings/day. The difference between the two risk estimates represented a risk attributable to consumption of sugar sweetened beverages. Using the risk estimates, sampling weights, and a population size, we estimated the absolute numbers of events over 10 years, events attributable to consumption of sugar sweetened beverages (absolute risk reduction^44^), and population attributable fraction (the proportion of events attributable to consumption of sugar sweetened beverages). The estimation assumed causality and no change in individuals’ characteristics over time. Validation of 10 year risk prediction was performed in the US survey, in which we predicted diabetes prevalence in 2009-10 by using data collected in 1999-2000. Sensitivity analysis was performed to estimate the population attributable fraction by varying relative risks and accounting for uncertainty. Further details are presented in the supplementary information.

## Results

After the removal of duplicates, we identified 1937 articles, reviewed 33 in full text, and identified 21 articles of 16 cohorts as being eligible for this meta-analysis (see supplementary figure S1). We obtained unpublished information on one cohort from a publication that did not meet eligibility criteria (see supplementary table S1).^22^ Finally, we evaluated data from 17 cohorts (table 1)^11^ ^39^ ^40^ ^41^ ^49^ ^50^ ^51^ ^52^ ^53^ ^54^ ^55^ ^56^ ^57^ ^58^ ^59^ ^60^ ^61^ ^62^ ^63^ ^64^ ^65^ comprising 38 253 cases of type 2 diabetes over 10 126 756 person years in total. No study or publication was funded by industry.

**Table 1 tbl1:** Characteristics of prospective cohort studies included in meta-analysis on associations between consumption of sugar sweetened beverages, artificially sweetened beverages, and fruit juice and incidence of type 2 diabetes

Cohort, country*	Baseline years	Median follow-up (years)	No	Age range or mean age (years)	Men (%)	Mean BMI	Beverage consumption†			Incident type 2 diabetes‡	
							Method of assessment	Types		Method of ascertainment	No of cases (rate/1000)‡
FMCHES, Finland^53^	1966-72	13.7	4304	40-69	53.1	26.5	Diet history	SSB		Records	175 (3.0)
NHS I, USA^41 51^	1984	21.1	74 513	40-69	0	23.6	FFQ†	SSB, ASB, FJ		Self report	7300 (4.6)‡
KIHD, Finland^54^	1984-89	18.9	2481	42-60	100	26.8	4 day diet record	SSB§		Records, biomarkers	506 (10.8)
CARDIA, USA^55 56^¶	1985-86	18.8	2160	18-30	46.5	24.5	Diet history†	SSB, ASB, FJ		Self report, records, biomarkers	174 (4.3)‡
HPFS, USA^41 49 50^	1986	19.3	40 290	40-75	100	25.5	FFQ†	SSB, ASB, FJ		Self report	3229 (4.2)‡
Iowa WHS, USA^62^¶	1986	10.7	31 489	55-69	0	27.0	FFQ	SSB, FJ		Self report	999 (3.0)‡
ARIC men, USA^64^	1987-89	7.5	5414	45-64	100	27.2	FFQ	SSB‡		Self report, biomarkers	718 (17.7)
ARIC women, USA^64^	1987-89	7.7	6790	45-64	0	27.2	FFQ	SSB‡		Self report, biomarkers	719 (13.8)
JPHC men, Japan^52^¶	1990	9.8	12 137	40-59	100	23.5	FFQ†	SSB, FJ		Self report	397 (3.3)‡
JPHC women, Japan^52^¶	1990	9.9	15 448	40-59	0	23.5	FFQ†	SSB, FJ		Self report	279 (1.8)‡
FOS, USA^65^	1991	12.1	2736	54.2	45.5	26.7	FFQ†	SSB, ASB, FJ		Records, self report, biomarkers	303 (9.1)
NHS II, USA^41 60 61^	1991	18.4	90 423	24-44	0	24.4	FFQ†	SSB, ASB, FJ		Self report	5121 (3.1)‡
EPIC-InterAct, eight European countries^11^	1991-98	11.7	27 058	52.4	37.8	26.0	FFQ	SSB, ASB, FJ		Records, biomarkers‡	11 684 (2.9)
E3N, France^40^¶	1993	12.4	48 985	52.8	0	22.8	Diet history	SSB, ASB, FJ		Records	1054 (1.7)
SCHS, Singapore^57^¶	1993-98	5.7	43 580	45-74	42.9	23.0	FFQ	SSB, FJ		Self report, records, biomarkers	2250 (9.0)‡
Black WHS, USA^58^	1995	7.7	43 960	21-69	0	27.6	FFQ	SSB, ASB, FJ		Self report	2550 (7.5)‡
HIPOP-OHP, Japan^63^¶	1999	3.4	6121	19-69	78.9	22.6	FFQ	SSB, FJ		Self report, records, biomarkers	212 (10.2)
MESA, USA^39^	2000-02	5.8	5011	45-84	47.4	27.9	FFQ	SSB, ASB		Self report, records, biomarkers	413 (14.3)
Occupational cohort, Japan^59^	2003	5.5	2037	35-55	100	23.3	FFQ	SSB, ASB, FJ		Records, biomarkers	170 (15.1)

ASB=artificially sweetened beverages; ARIC=Atherosclerosis Risk in Communities Study; CARDIA=Coronary Artery Risk Development in Young Adults Study; EPIC=European Prospective Investigation into Cancer and Nutrition Study; FFQ=food frequency questionnaires; FJ=fruit juice; FMCHES=Finnish Mobile Clinic Health Examination Survey; FOS=Framingham Offspring Study; HIPOP-OHP=High-risk and Population Strategy for Occupational Health Promotion Study; HPFS=Health Professional Follow-up Study; JPHC=Japan Public Health Center-based Prospective Study; KIHD=Kuopio Ischaemic Heart Disease Risk Factor Study; MESA=Multi-Ethnic Study of Atherosclerosis; NHS=Nurses’ Health Study; SCHS=Singapore Chinese Health Study; SSB=sugar sweetened beverages; WHS=Women’s Health Study.

*Ordered in years of baseline assessments. Numbers represent citations. ARIC and JPHS reported results stratified by sex. In meta-analysis, sex stratified estimates were aggregated in advance.

†Diets were assessed repeatedly during follow-up and incorporated in longitudinal analysis. JPHC measured repeatedly but used baseline FFQ only.

‡Biomarkers included any of fasting glucose, two hour glucose by oral glucose tolerance test, and glycated haemoglobin. Records included medical records or other records from registry, not including self reported information. Studies ascertaining cases of type 2 diabetes by self report involved uncertainty in ascertainment, and thus numbers of cases were revised by a positive predictive value (proportion of verified cases among self reported cases) (see supplementary information and table S3). The EPIC-InterAct study adopted different methods across participating cohorts, in which no cohort used self reported diagnosis only.

§In ARIC and FOS analyses, sugar sweetened and artificially sweetened beverages were combined. In KIHD, the article described fruit juices, but treated them as sugar sweetened beverages, because more than 90% of fruit juice consumed in Finland in 1980s and 1990s was sweetened with sugars (confirmed by the authors).

¶Considered as having potential bias (see supplementary information and table S2). In each of CARDIA and JPHC, distinct analytic approaches were undertaken in different publications by the same authors. Iowa WHS reported results only in a conference abstract. E3N presented prospective associations adjusted for crude categorical variables for body mass index, which could cause substantial residual confounding. SCHS presented results without classification between 100% fruit juice and sugar sweetened fruit juice and had a likelihood of attrition bias by loss of follow-up by deaths (15%). HIPOP-OHP lost 31% of participants during follow-up, which could cause bias due to differential misclassification.

The quality of the studies has been examined (see supplementary table S2). Methods of assessing diets and ascertaining type 2 diabetes and validity of these measurements varied across studies (table 1 and supplementary table S3). We identified potential bias in the quantitative results for six cohorts based on at least one of the following: publication of a conference abstract only,^62^ exclusion of participants lost during follow-up,^57^ ^63^ likelihood of substantial residual confounding,^40^ and no separation between fruit juice and sugar sweetened beverages (fruit drinks) or between sugar sweetened beverages and artificially sweetened beverages.^57^ ^64^ Selective reporting might exist in some studies,^39^ ^52^ ^55^ ^58^ ^62^ ^64^ but it was unlikely to cause bias—for example, reporting only non-quantitative results for sugar sweetened beverages in a study mainly on artificially sweetened beverages.^39^ Other potential sources of bias were detected, but we did not consider them to be substantially influential on overall bias in each study, partly based on results of sensitivity analyses. No study assessed subtypes of sugar sweetened beverages, artificially sweetened beverages, or fruit juice, except one that separated beverages by caffeine content.^49^

Confounding was likely to exist in all of the studies. As would be expected, consumers of artificially sweetened beverages tended to be overweight or obese or hypertensive.^11^ ^39^ ^50^ ^59^ ^66^ In longitudinal analysis, all studies statistically adjusted for potential confounders, such as sociodemographic variables, clinical factors (family history of diabetes or prevalent diseases), and lifestyle factors, including diet (see supplementary table S4). None of these factors was identified as a single cause of confounding, according to studies assessing influence of potential confounding in different regression models.^11^ ^41^ ^49^ ^50^ ^51^ ^54^ ^59^ ^61^ ^63^ ^64^ ^65^ However, a combination of multiple factors was likely to cause confounding (table 2 and supplementary table S4). After adjustment for multiple potential confounders, the relative risk for sugar sweetened beverages was attenuated from 1.25 to 1.18 (32% change), and for artificially sweetened beverages from 1.48 to 1.25 (43%). By contrast, the point estimate for fruit juice was shifted upwards, from 0.97 to 1.05.

**Table 2 tbl2:** Associations between consumption of sugar sweetened beverages, artificially sweetened beverages, and fruit juice and incident type 2 diabetes: meta-analysis of prospective cohort studies

Beverages (No of cohorts) and models of meta-analysis*	Not adjusted for adiposity†			Adjusted for adiposity†	
	Relative risk (95% CI)	I^2^ (%)		Relative risk (95% CI)	I^2^ (%)
**Sugar sweetened beverages (n=17)**					
Meta-analysis, crude:	1.25 (1.14 to 1.37)	89		—	—
+multivariable adjusted	1.18 (1.09 to 1.28)	89		1.13 (1.06 to 1.21)	79
+calibration for information bias	1.43 (1.20 to 1.70)	86		1.28 (1.12 to 1.46)	73
+calibration for publication bias	1.42 (1.19 to 1.69)	85		1.27 (1.10 to 1.46)	73
**Artificially sweetened beverages (n=10)**					
Meta-analysis, crude:	1.48 (1.35 to 1.62)	85		—	—
+multivariable adjusted	1.25 (1.18 to 1.33)	70		1.08 (1.02 to 1.15)	64
+calibration for information bias	2.13 (1.57 to 2.88)	72		1.29 (1.08 to 1.54)	50
+calibration for publication bias	1.81 (1.33 to 2.47)	76		1.22 (0.98 to 1.52)	64
**Fruit juices (n=13)**					
Meta-analysis, crude:	0.97 (0.90 to 1.06)	79		—	
+multivariable adjusted	1.05 (0.99 to 1.11)	58		1.07 (1.01 to 1.14)	51
+calibration for information bias	1.06 (0.98 to 1.14)	49		1.10 (1.01 to 1.20)	29
+calibration for publication bias	Not detected	—		Not detected	—

*Number of cohorts varied slightly by models (see fig 1). Crude meta-analysis pooled estimates without any adjustment. Multivariable adjusted estimates were based on meta-analysis of estimates adjusted for demographic and lifestyle covariates (see supplementary table S4 for details). Calibration for information bias accounted for within person variation for dietary consumption and imprecise ascertainment of self reported diabetes. Calibration for publication bias was carried out, if indicated in trim and fill analysis.

†Relative risk and 95% confidence intervals per serving/day before and after adjustment for adiposity. All relative risks were significant (P<0.05), except for artificially sweetened beverages after adjustment for publication bias (P=0.07).

### Beverage consumption and type 2 diabetes

Table 2 summarises the findings from meta-analysis. Higher consumption of sugar sweetened beverages by one serving per day was associated with an 18% greater incidence of type 2 diabetes (95% confidence interval 8.8% to 28%; I^2^=89%) before adjustment for adiposity (fig 1 and table 2). When adjusted for potential mediation and confounding by adiposity, the association was attenuated, with the incidence increased by 13% per serving/day (5.8% to 21%; I^2^=79%). In the analysis of artificially sweetened beverages, in which adiposity was unlikely to be a mediator, higher consumption of artificially sweetened beverages by one serving per day was associated with a 25% greater incidence of type 2 diabetes (95% confidence interval 18% to 33%; I^2^=70%) before adjustment for adiposity. After adjustment, the estimate of 25% greater incidence was attenuated to 8% (2.1% to 15%).

**Figure fig1:**
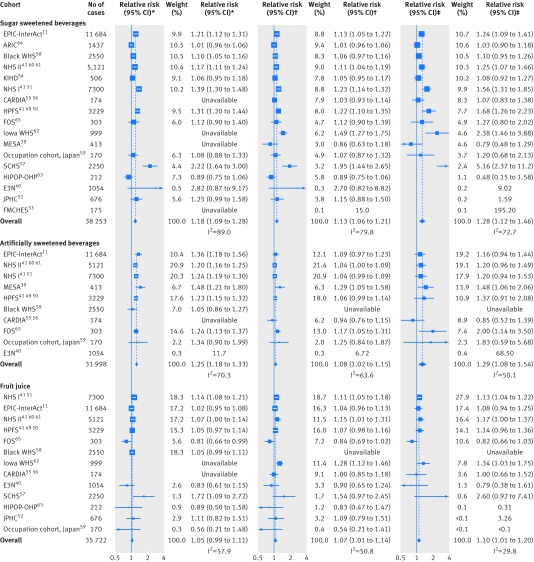
**Fig 1** Prospective associations of beverage consumption with incident type 2 diabetes: random effects meta-analysis. *Unadjusted for adiposity. †Adjusted for adiposity. ‡Adjusted for adiposity and within person variation. Cohorts were ordered by weights in the most adjusted model. Estimates with 95% confidence intervals greater than 10 are not presented. Supplementary table S4 summarises the covariates adjusted for in each study

In the analysis of fruit juice, the influence of adjustment for adiposity was in the direction opposite to that of the analysis for sugar sweetened beverages and artificially sweetened beverages. The association of fruit juice consumption with incident type 2 diabetes was strengthened after adjustment for adiposity measures. Higher consumption of fruit juice by one serving/day was associated with a 7% greater incidence of type 2 diabetes (95% confidence interval 0.8% to 14%).

Each of the beverages showed significant non-linear associations (P>0.05) (supplementary figure S2). Calibration for within person variation strengthened the association between each type of beverage and incident type 2 diabetes (fig 1 and table 2). For example, the relative risk per one serving/day of sugar sweetened beverages was strengthened, from 1.13 (95% confidence interval 1.06 to 1.21) to 1.28 (1.12 to 1.46). Estimates for artificially sweetened beverages were strengthened similarly. The influence was small for fruit juice, where the relative risk was shifted only slightly, from 1.07 (1.01 to 1.14) to 1.10 (1.01 to 1.20).

### Sensitivity analysis and quality of evidence

None of the study specific factors evaluated could explain heterogeneity of results for sugar sweetened beverages and artificially sweetened beverages (P>0.1) (see supplementary table S5). Exploratory meta-regression produced an I^2^ of 23.4% for sugar sweetened beverages and of 67.8% for artificially sweetened beverages, adjusted for population demographics (age, sex, country, incidence), body mass index, follow-up duration, and measures of study quality. The results for fruit juice varied by study design. While studies assessing self reported type 2 diabetes only showed a positive association, the significant association disappeared in studies ascertaining the incidence of type 2 diabetes by medical records or by blood glucose or glycated haemoglobin level (relative risk 1.08, 0.97 to 1.20; P for heterogeneity=0.008). Additionally, studies with repeated measures of diets supported a null finding (0.98, 0.86 to 1.11; P for heterogeneity=0.068). These factors of study design explained heterogeneity of the association, reducing I^2^ from 29% to 0%. Demographic variables and body mass index did not explain heterogeneity (P>0.14 each), whereas sugar sweetened beverages, artificially sweetened beverages, and fruit juice were not significantly associated with type 2 diabetes in studies recruiting more men than women or conducted in Asia, with a fewer number of studies than in the main analysis (supplementary table S5).

Publication bias was not evident by Egger’s test (P>0.05), except for fruit juice (P=0.03), where estimates with the greater precision showed stronger associations (fig 2). Trim and fill indicated publication bias for both sugar sweetened and artificially sweetened beverages (table 2). In particular, publication bias could influence inference for artificially sweetened beverages. With adjustment for adiposity, the relative risk per one serving/day of artificially sweetened beverages was 1.29 (1.08 to 1.54) before calibration for publication bias and 1.22 (0.98 to 1.52) after calibration (table 2).

**Figure fig2:**
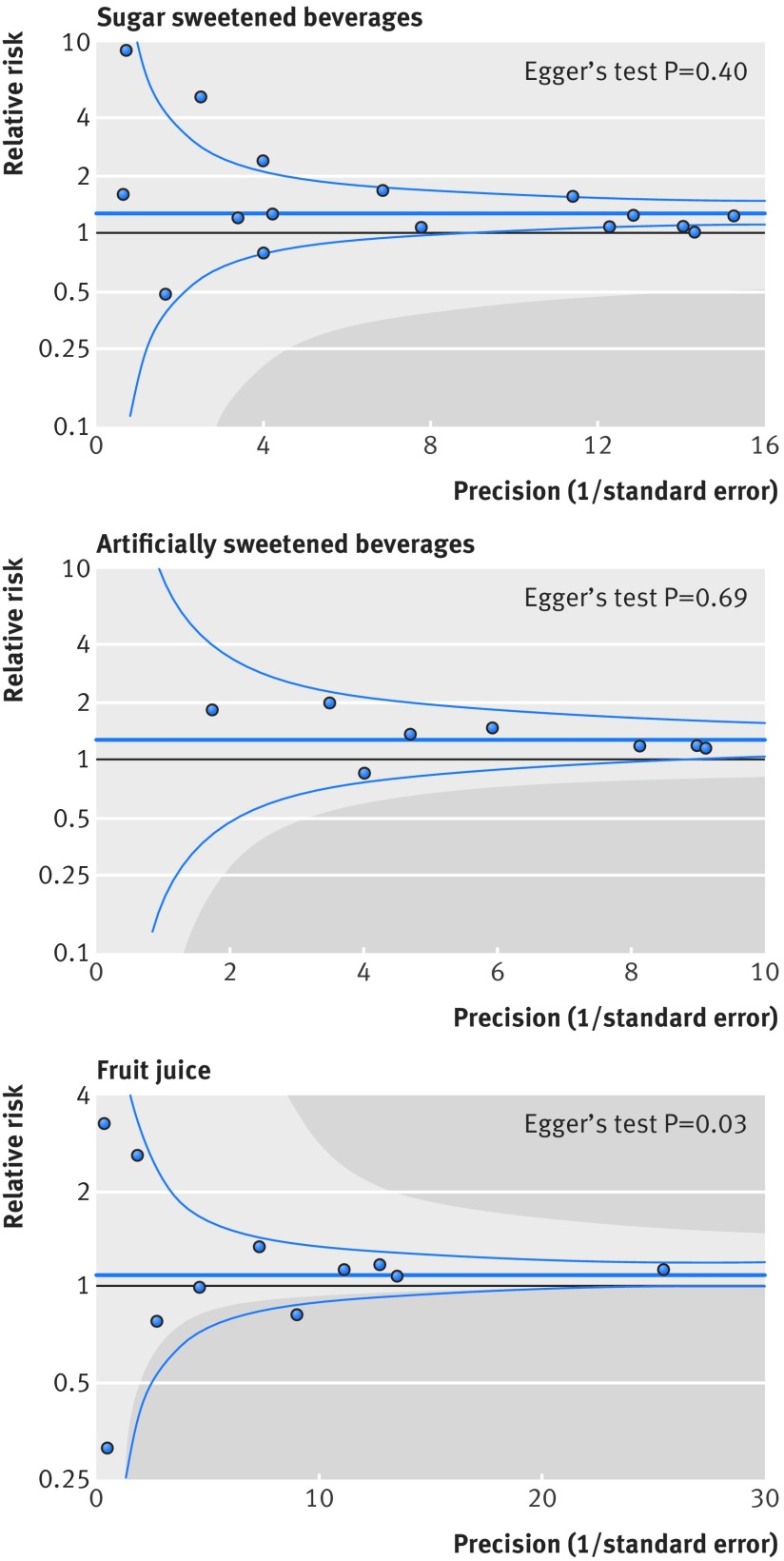
**Fig 2** Funnel plot for associations of sugar sweetened beverages, artificially sweetened beverages, and fruit juice with incident type 2 diabetes. Dots represent point estimates plotted over precision measures (1/standard error). Estimates outside each panel are not presented. Horizontal lines represent summary estimates and 95% confidence intervals across precision. Shaded areas represent any of a single estimate that, if included, would make the summary estimate insignificant (P>0.05). P values by Egger’s test are presented: for fruit juice, estimates with greater precision indicated stronger positive association

Contour enhanced funnel plots indicated that the findings for artificially sweetened beverages and fruit juice were not stable (fig 2). For example, if a study with a relative risk of 0.5 contributed to the meta-analysis, associations of artificially sweetened beverages and fruit juice with type 2 diabetes incidence would not be significant. In influence analysis, positive associations persisted for sugar sweetened beverages and artificially sweetened beverages (see supplementary figure S3), whereas a significant result of fruit juice was not seen after excluding any single studies supporting the positive association.

The results varied little by methodological assumption (see supplementary table S6), using estimates per 250 mL/day (median of 17 studies) rather than per serving/day; excluding studies with a high risk of potential bias or with relatively large within person dietary variability; and incorporating uncertainty of within person variability and precision of type 2 diabetes diagnosis. When we examined the potential influence of residual confounding by measured adiposity, bias towards the null appeared substantial for artificially sweetened beverages (see supplementary figure S4). Under a realistic assumption of a correlation of 0.80 between measured and true adiposity,^36^ the association for sugar sweetened beverages was attenuated by 26% (relative risk 1.20, 1.04 to 1.38), for artificially sweetened beverages was attenuated by 96% (1.01, 0.81 to 1.25), and for fruit juice was strengthened by 19% (1.12, 1.03 to 1.22).

We rated the quality of evidence for sugar sweetened beverages, artificially sweetened beverages, and fruit juice. The evidence for sugar sweetened beverages was rated as being of moderate quality. The main finding rejected the null hypothesis and was likely to have a small degree of heterogeneity unexplained, a dose-response relation, and robustness against potential bias or limitations including publication bias. We rated the evidence for artificially sweetened beverages and fruit juice to be of low quality. Findings for artificially sweetened beverages were likely to have publication bias and residual confounding; and for fruit juice, the positive association was not stable and varied by study design.

### Type 2 diabetes risk attributable to sugar sweetened beverages

Overall, sugar sweetened beverages were consumed by 54.4% of people in the USA and 49.4% in the UK. Of a total population, the mean consumption of sugar sweetened beverages was 284 (SD 412) g/day in the USA and 114 (SD 157) g/day in the UK (fig 3 and supplementary table S7). Absolute event rates over 10 years from 2010 were estimated to be 11.0% in the USA (20.9 million events) and 5.8% in the UK (2.6 million events). Assuming a causal effect of consumption of sugar sweetened beverages partly mediated by obesity status (adiposity unadjusted), consumption in the USA would result in 2.6 million excess events of type 2 diabetes over 10 years (population attributable fraction 11.9%, 95% confidence interval 7.4% to 16.5%); and 126 000 excess events in the UK (population attributable fraction 4.9%, 3.0% to 7.2%). Assuming a causal effect of consumption of sugar sweetened beverages independent of obesity status (adiposity adjusted), consumption would result in 1.8 million excess events in the USA (population attributable fraction 8.7%, 3.9% to 12.9%) and 79 000 excess events in the UK (population attributable fraction 3.6%, 1.7% to 5.6%). Younger adults and men would have greater numbers of type 2 diabetes events related to consumption of sugar sweetened beverages than older adults and women, respectively (fig 3 and supplementary table S7). The greater the uncertainty accounted for, the lesser the precision was computed (see supplementary figure S5). For example, if I^2^=50%, the population attributable fraction of 11.9% in the USA would have a 95% confidence interval of 5.2% to 18.3%, but remained significant from 0 for the relative risks we estimated. 

**Figure fig3:**
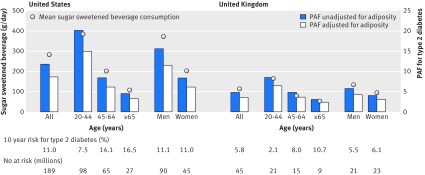
**Fig 3** Consumption of sugar sweetened beverages and population attributable fraction (PAF) for type 2 diabetes in the United States and the United Kingdom. Circles represent mean of consumption of sugar sweetened beverages (left axis) and each bar represents PAF (%) for type 2 diabetes due to consumption of sugar sweetened beverages (right axis). Absolute event rates over 10 years were 11.0% in the USA (20.9 million events) and 5.8% in the UK (2.6 million events) (see supplementary table 7)

## Discussion

In this systematic review and meta-analysis, we have produced summary evidence that habitual consumption of sugar sweetened beverages, artificially sweetened beverages, and fruit juice was prospectively associated with incident type 2 diabetes, independently of adiposity. Sensitivity analyses consistently supported the positive association of sugar sweetened beverages with incident type 2 diabetes. In contrast, the association between artificially sweetened beverages or fruit juice and incident type 2 diabetes was less evident. For artificially sweetened beverages, potential publication bias and residual confounding were likely to exist. For fruit juice, the finding seemed to be unstable and was sensitive to study design. Under assumption of causality for the association of consumption of sugar sweetened beverages with incidence of type 2 diabetes, we provided efficacy estimates that over 10 years two million type 2 diabetes events in the USA and 80 000 in the UK would be related to consumption of sugar sweetened beverages.

### Strengths and limitations of this review

This study has limitations typical of observational studies and meta-analysis. Residual confounding could exist.^37^ Confounding by socioeconomic and dietary factors was not detected to be strong in published studies. However, measures of these variables were likely to involve errors such that residual confounding persisted in individual studies and our meta-analysis. Additionally, lifestyle factors and adiposity could change over time. The time varying characteristics might not be random and could result in bias in an unknown direction. Reverse causality could also exist because of unmeasured comorbid conditions and health consciousness that might alter consumption of beverages, particularly artificially sweetened ones, and risk of type 2 diabetes. Weakness of meta-analysis includes the exclusion of eligible cohorts for lack of information. Our meta-analysis included statistical approximation that might involve errors. For example, we derived dose-response estimates partly from categorical estimates and odds ratios. Without such approximations, analysis standardised across different cohorts is of future interest to characterise associations between various beverages and risks of type 2 diabetes. Finally, assessments of bias and quality of evidence involved subjectivity, although we objectively examined the influence of potential bias in tests for heterogeneity and sensitivity analyses.

One strength of this study was in estimating population attributable fraction for risks of type 2 diabetes related to consumption of sugar sweetened beverages in the USA and UK, using data on beverage consumption for each individual. While a few studies estimated how much taxation on sugar sweetened beverages influenced risk of obesity and type 2 diabetes,^12^ ^13^ ^14^ no study examined the population impact by combining estimates for consumption of sugar sweetened beverages in multiple populations, predicted the risk of type 2 diabetes by using a validated algorithm, or provided quantitative evidence on the association of consumption of sugar sweetened beverages with incidence of type 2 diabetes. However, the population attributable fraction was limited in precision owing to underlying relative risks and uncertainty in generalisability. The other limitations are that we estimated the population attributable fraction under the assumption of causality, although it has not been established; and the assumption that there would be no change over time in lifestyle associated with consumption of sugar sweetened beverages. Future work should seek to improve precision of evidence and to characterise efficacy and effectiveness of policy interventions for different populations. Generalisability should be explored—for example, for populations in Central and South America with the highest recorded per capita sales of sugar sweetened beverages in the world; and China and India where the highest prevalence of type 2 diabetes is expected.^1^ ^67^ To minimise limitations typical of observational research and understand effectiveness of a policy intervention in different populations, future research should also include randomised trials examining people’s health and behaviours and informing effectiveness.

### Interpretation in relation to other studies

Other quantitative reviews have been published recently.^4^ ^5^ ^9^ None of them quantified the population attributable fraction. One meta-analysis evaluated the influence of adiposity on the association of sugar sweetened beverages and type 2 diabetes based on three studies, but not artificially sweetened beverages or fruit juice.^5^ For sugar sweetened beverages, we evaluated a greater number of type 2 diabetes cases (38 285 *v* 19 054) and studies (17 *v* 3), including bias assessments and sensitivity analyses. For fruit juice and artificially sweetened beverages, we evaluated a greater number of studies than previous work (9 *v* 4 and 12 *v* 4, respectively) and drew conclusions based on assessment of the influences of adiposity and potential bias that were found to be important.

The plausibility of our findings deserves discussion. Detrimental effects of sugar sweetened beverages independent of obesity may exist. Sugars in sugar sweetened beverages acutely increased blood glucose levels and have a high glycaemic index (80 to 110/100 of white bread), a risk factor for type 2 diabetes.^68^ ^69^ Fructose in sugar sweetened beverages promotes hepatic lipogenesis and further insulin resistance.^2^ Effects of caramels for colouring beverages,^70^ caffeine,^49^ ^71^ phosphoric acid,^72^ and other constituents may also exist. These non-glycaemic effects may be present in artificially sweetened beverages, if such beverages truly increase the risk of type 2 diabetes. Artificially sweetened beverages might have effects on hormones, microbiota, and taste preference, but evidence for these remains weak.^6^ ^73^ ^74^ Adverse effects of fruit juice would be present because of its moderately high glycaemic index (50-80).^68^ Healthful constituents may exist but decrease during processing.^7^ ^75^ This explains why our finding was discordant with the inverse association of consuming fruits as food with type 2 diabetes.^76^

Our analysis indicated possible publication bias for the associations between artificially sweetened beverages and type 2 diabetes. The bias toward a false positive finding would be plausible according to existing public interest over the health effects.^6^ ^77^ The finding at least underscores potential low quality of evidence and the need for cautious interpretation. Residual confounding in the finding for artificially sweetened beverages is also plausible because adults at high risk of type 2 diabetes preferentially consumed more artificially sweetened beverages.^5^ ^6^ ^8^ ^10^ ^11^ ^36^ Confounding in the opposite direction in the finding for fruit juice is also plausible because leaner adults at lower risk of type 2 diabetes consumed more fruit juice.^10^ ^11^ These observations provide research and clinical implications for better understanding of health seeking behaviours related to beverage consumption.^6^ ^78^

### Clinical and public health implications

Although causality has not been established, our findings and available evidence indicate a benefit of reducing the consumption of sugar sweetened beverages for the primary prevention of type 2 diabetes. In the same context, our findings also imply that consumption of artificially sweetened beverages or fruit juice is not likely to reduce the risk of type 2 diabetes and, thus, not suitable as a healthy option. None the less, the lower caloric intake of artificially sweetened beverages may be of clinical benefit in obese or overweight adults by helping to reduce body weight.^6^ ^78^ This effect on body weight should be considered separately from our study, which could not rule out the effect of body weight on beverage consumption.^8^ Additionally, clinical applications of our finding deserve further appraisal about the effects of altering beverage consumption on changes in lifestyle behaviours and on risks of other clinical outcomes.^3^ ^6^ ^8^

Our findings have strong public health implications. Despite the limitations of this review, the current consumption of sugar sweetened beverages was estimated to cause approximately two million excess events of type 2 diabetes in the USA and 80 000 in the UK over 10 years. This could cost nearly £12.0bn in the USA and £206m in the UK ($9800 in the USA and $3994 in the UK per patient,^67^ $1 (£0.65; €0.91 as of 7 July 2015). In future, our work on efficacy should be extended to that on effectiveness to identify needs for interventions. In addition to observational evidence, trial evidence should be available, accounting for the effects on cardiometabolic health and lifestyle change associated with a possible intervention.^8^ ^78^ Despite a population attributable fraction of no more than 20%, effectiveness should be evaluated for different populations, as an estimated 592 million adults globally will have type 2 diabetes in 2035.^1^ ^67^ Additionally, the average population attributable fraction of no more than 20% confirms the importance of modifying multiple lifestyle risk factors rather than a single dietary component, for the primary prevention of type 2 diabetes. For artificially sweetened beverages and fruit juice, our findings inform little benefit of using them as an alternative to sugar sweetened beverages. In addition, fruit juice consumption should not be a part of dietary recommendations for greater consumption of fruits and vegetables, as suggested to limit fruit juice consumption among children.^7^ ^79^

### Conclusions

Observational cohort studies support that consumption of sugar sweetened beverages is associated with incident type 2 diabetes, and independently of adiposity. This finding was stable in sensitivity analyses assessing influence of population characteristics, potential residual confounding, and publication bias. By contrast, although artificially sweetened beverages and fruit juice showed a positive association with incident type 2 diabetes, the quality of evidence is limited by potential bias and heterogeneity by study design. Although causality has not been established and precision needs to be improved, this study informs the potential efficacy of reducing the consumption of sugar sweetened beverages in a contemporary population. Moreover, findings support that neither artificially sweetened beverages nor fruit juice are suitable alternatives to sugar sweetened beverages for the prevention of type 2 diabetes.

**What is already known on this topic**

Current evidence is limited to answer whether or not consumption of sugar sweetened beverages, artificially sweetened beverages, and fruit juice is associated with risk of diabetes after adjustment for obesity status
Despite in the debate of a policy intervention to reduce consumption of sugar sweetened beverages, no study has estimated how many incident cases of diabetes may be caused by consuming sugar sweetened beverages

**What this study adds**

Habitual consumption of sugar sweetened beverages was positively associated with incidence of type 2 diabetes, independently of obesity status
Under an assumption of causality, consumption of sugar sweetened beverages may be linked to 4-13% of type 2 diabetes incidence in the United States and 2-6% in the United Kingdom over 10 years, 2010-20
Artificially sweetened beverages and fruit juice were both positively associated with incident type 2 diabetes, but likely to involve bias, and seemed not to be healthy options for the prevention of type 2 diabetes

## Notes

The following contributors provided additional information: Emilie Rossignol, Guy Fagherazzi, Françoise Clavel-Chapelon, and Beverley Balkau, Center for Research in Epidemiology and Population Health, Villejuif Cedex, France; Tomonori Okamura, Department of Preventive Medicine and Public Health, Keio University School of Medicine, Tokyo, Japan, and Hirotsugu Ueshima, Department of Health Science and Center for Epidemiologic Research in Asia; Sari Voutilainen, Institute of Public Health and Clinical Nutrition, Kuopio, Finland; Frank B Hu, Harvard T H Chan School of Public Health, Boston, Massachusetts, USA; Manabu Sakurai, Department of Epidemiology and Public Health, Kanazawa Medical University, Uchinada, Ishikawa, Japan; Paul F Jacques, Nicola M McKeown, and Ma Jiantao, Human Nutrition Research Center on Aging, Tufts University, Boston, Massachusetts, USA.

Contributors: FI and NGF conceived the study. FI, LO’C, YZ, and NGF designed the study. FI, LO’C, and YZ undertook the literature search and extracted data. FI, JM, YH, and SNB did data analysis. FI, LO’C, YZ, and NGF interpreted data. FI developed the first draft. FI as guarantor accepts full responsibility for the work and the conduct of the study, had access to the data, and controlled the decision to publish. All authors provided critical comments and approved the final version. The sponsor of the study had no role in design and conduct of the study; collection, management, analysis, and interpretation of the data; preparation, review, or approval of the manuscript; and decision to submit the manuscript for publication. The corresponding author had full access to all the data in the study and had final responsibility for the decision to submit for publication.

Ethical approval: Not required.

Data sharing: A protocol and data for tables and figures are available on request.

Transparency: The guarantor (FI) affirms that the manuscript is an honest, accurate, and transparent account of the study being reported; that no important aspects of the study have been omitted; and that any discrepancies from the study as planned have been explained as post hoc analysis. Data sharing is available from the corresponding author.

## Notes

Cite this as: *BMJ* 2015;351:h3576
