# Supplementary material for: Consumption of sugar sweetened beverages, artificially sweetened beverages, and fruit juice and incidence of type 2 diabetes: systematic review, meta-analysis, and estimation of population attributable fraction
Source: BMJ. 2015 Jul 21;351:h3576. doi: 10.1136/bmj.h3576 (PMC4510779; doi:10.1136/bmj.h3576)
Supplement: Supplementary file 1 [file imaf023070.ww1_default.pdf]

Supplementary materials for "Prospective associations of consumption of sugar-sweetened beverages, artificially sweetened beverages, and fruit juice with type 2 diabetes: a systematic review, meta-analysis, and estimation of population attributable fraction"

Fumiaki Imamura, Laura O'Connor, Zheng Ye, Jaakko Mursu, Yasuaki Hayashino, Shilpa N Bhupathiraju, Nita G. Forouhi

Contents

Figure S1. Systematic identification of published literature on beverage consumption and type 2 diabetes. 2
Table S1. Studies reviewed in full text for eligibility and included or excluded for meta-analysis. 3
Table S2. Quality assessment of cohort studies included in meta-analysis of sweet beverages and incidence of type 2 diabetes. 6
Table S3. Validity measures of beverage consumption and incident type 2 diabetes. 7
Table S4. Assessment of potential confounders in the studies included in the meta-analysis of beverage consumption and type 2 diabetes. 9
Figure S2. Non-linear associations of consuming sugar-sweetened beverages, artificially-sweetened beverages, and fruit juice with incident type 2 diabetes. 10
Figure S3. Influence analysis for the prospective associations of consuming sugar-sweetened beverages, artificially-sweetened beverages, and fruit juice with incident type 2 diabetes. 11
Table S5. Potential sources of heterogeneity for the prospective associations of consuming sweet beverages with incident type 2 diabetes. 12
Table S6. Associations of consuming sweet beverages with incident type 2 diabetes by sensitivity meta-analysis. 13
Figure S4. Assessment of prospective association of consuming artificially sweetened beverages with incident type 2 diabetes after adjustment for assumed misclassification of adiposity measurements. 14
Table S7. The number of type 2 diabetes events preventable over 10 years from 2010 by eliminating sugar-sweetened beverage consumption among adults in the United States and in the United Kingdom. 15
Figure S5. Population attributable fraction (PAF) for different degrees of associations between consumption of sugar sweetened beverages (SSB) and incidence of type 2 diabetes in the United States and the United Kingdom: Sensitivity analyses. 16
Supplementary Text 17
- Search Strategy 17
- Identification of studies and contact to authors 17
- Quality assessment 17
- Adjustment for within-person variation of beverage consumption 18
- Adjustment for precision of incident type 2 diabetes 19
- Assessment of heterogeneity 19
- Sensitivity analysis 20
- Sensitivity analysis for residual confounding 20
- Estimation of type 2 diabetes events over 10 years from 2010 attributable to consumption of sugar-sweetened beverages in the United States and in the United Kingdom. 20

References of Supplementary Materials 22

Correspondence:
Fumiaki Imamura, MS PhD
MRC Epidemiology Unit, Institute of Metabolic Science,
University of Cambridge School of Clinical Medicine, Box 285,
Cambridge Biomedical Campus, Cambridge, CB2 0QQ, United Kingdom
Tel: +44 (0) 1223 769208, Fax: +44 (0) 1223 330316, Email: fumiaki.imamura@mrc-epid.cam.ac.uk

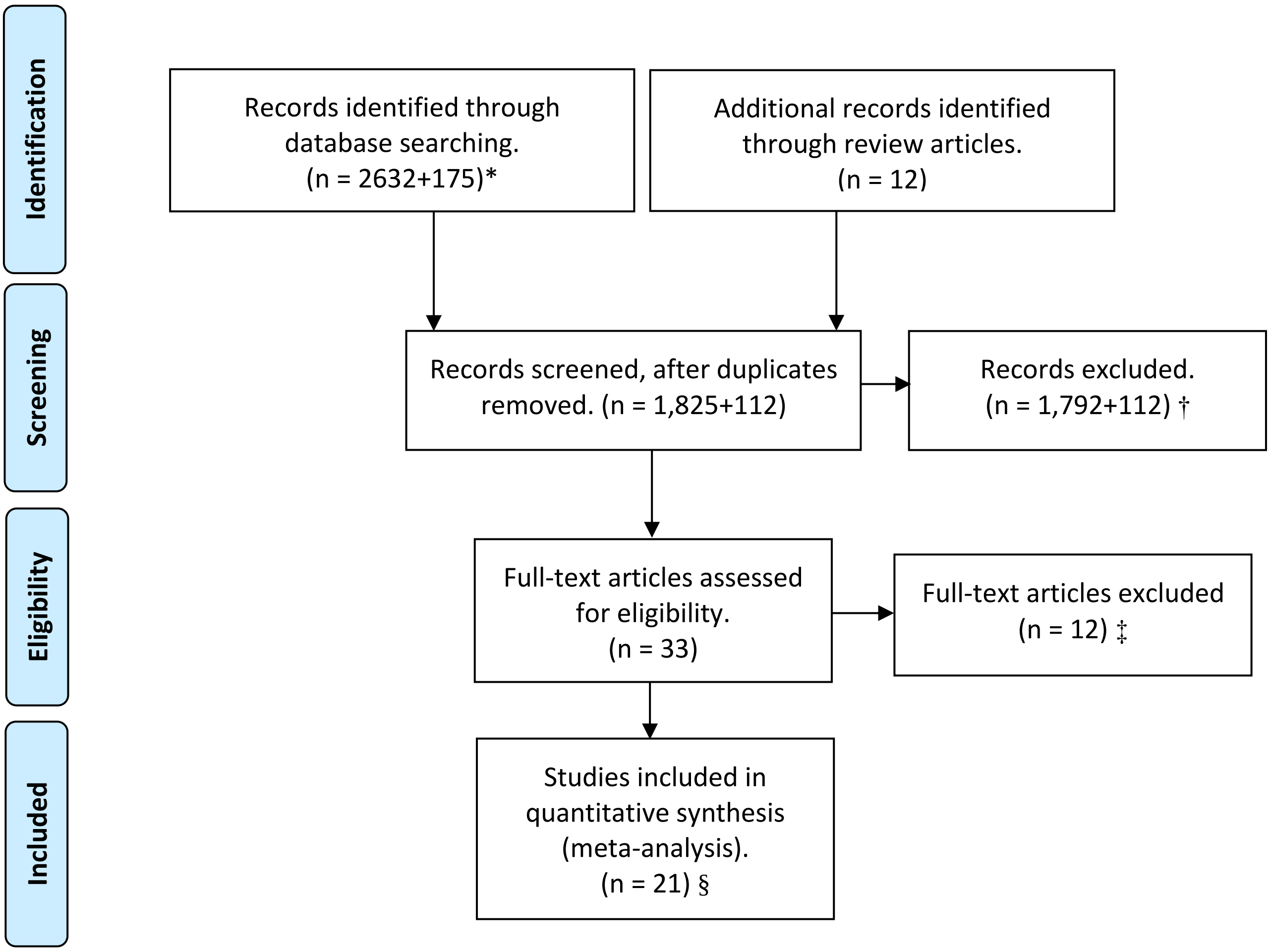

Figure S1. Systematic identification of published literature on beverage consumption and type 2 diabetes. Search terms are described in Supplementary Text. * Two values indicate search on 31 May 2013 and search on 10 February 2014. † Major reasons for exclusions for initial screening: main exposures were alcohol, coffee, or other dietary factors, rather than sweetened beverages or fruit juice; outcomes were not diabetes, either recruiting diabetes patients or assessing diabetes as covariate; studies were cross-sectional; studies recruited children; and publications are reviews, editorials, commentaries or other formats. ‡ See Table S1 for reasons for exclusion. § Seventeen cohorts, as a few cohorts published more than one article examining different beverages. One cohort met eligibility criteria after we obtained additional information.

Table S1. Studies reviewed in full text for eligibility and included or excluded for meta-analysis.*****

Cohort, country*	Results from full text review and author contact *	Decision for the present meta-analysis
**Identified as potentially eligible and reviewed †**		
BRHS, UK[1,2]<br>Cohort in Australia[3]<br>EBSHP, US.[4]	Ineligible, no information on any assessments of diets. We did not contact the authors.	Excluded.
EPIC-NL, Netherlands.[5]	Eligible, but the study was included in EPIC-InterAct.	Excluded.
Hisayama, Japan[6]	Ineligible. A diet and incident diabetes were assessed, but the authors confirmed no information on consumption of sweetened beverages and fruit juice.	Excluded.
SUN Study, Spain[7]<br>WHS, US[8,9]<br>PHS, US[9]<br>NIH-AARP, US[10]<br>D.E.S.I.R, France[11]	Eligible, but not included. Each cohort had information on dietary consumption and incident T2D. The authors could not respond to our request, because a resource was limited to conduct analyses we requested.	Excluded.
HIPOP-OHP, Japan[12]	Eligible, reported information of SSB consumption and incident T2D. After we contacted the authors, the authors provided sufficient information.	New unpublished estimates were used.
**Identified as eligible**		
FMCHES, Finland[13]	SSB and sugar-sweetened berry juices were reported separately. The authors did not respond to our request to combine the two.	Reported statistics on SSB were used.
NHS I, US[14,15]<br>HPFS, US[14,16,17]<br>NHS II, US[14,18,19]	Each cohort was censored at different time-point depending on types of beverages. Also, other publications from the cohorts indicate availability of data based on longer follow-up.	Analyses were updated using the censoring date in each dataset up to date (NHS, up to 2008; NHS II, 2011; and HPFS, 2010).
KIHDS, Finland[20]	Information was available in analyses excluding hyperglycaemic adults..	Updated analyses additionally including hyperglycaemic adults at risk of developing diabetes (little change in results).
CARDIA, US[21,22]	Eligible, evaluating beverage consumption and hyperglycaemia and having information on incident T2D. Positive responses were obtained, but new estimates were eventually not available.	Reported statistics were used. We used reported estimates for hyperglycaemia, accounting for the proportion of T2D cases (see Table S4).
Iowa WHS, US[23]	The publication was an abstract presented at a conference, thus not fully peer-reviewed and missing information needed.	Generic information on the cohort was obtained by another publication from Iowa WHS.[24] Exposure distributions of the adults were

		approximated by consumption observed in women in ARIC[25], considering similarity in chronological and demographic characteristics of women of the two cohorts.
FOS, US[26]	The original paper examined SSB and hyperglycaemia only. One author provided estimates needed for meta-analysis, evaluating each of sweetened beverages and fruit juice, by methods as previously reported.[27]	New estimates were used as provided.
ARIC, US[25]	Fruit juice, SSB and ASB were combined. We requested to separate them and update estimates matched with our objectives. We did not obtain any response.	Only reported statistics were used. The authors stated no change in results after adjustment for measures of adiposity (body-mass index and waist-to-hip ratio). ‡
JPHC, Japan[28]	Repeated measures were available in the cohort, but not used.[29] We requested analyses using them, but the authors decided not to do, being concerned of lack of peer-review of the specific methods.	Reported statistics based on 10-year follow-up were used. Repeated measures of diets were not used, although they could be used.[29,30]‡
EPIC-InterAct, eight European countries[31]	Statistics for each type of beverages and incident T2D before and after adjustment for measures of adiposity were available. The most recent data were analysed. Thus, no contact was attempted	Reported statistics were used.
E3N, France[32]	This cohort participates in EPIC-InterAct[31,33]. Two articles partly included the same adults. To avoid double-counts of overlapping adults, we requested analyses of 48,985 women after excluding 20,851 adults eligible for EPIC-InterAct. The authors responded to our request.	Estimates without overlap with InterAct were used.
SCHS, Singapore[34]	Availability of ASB was not clear. Fruit juice was evaluated with vegetable juice. We requested information for the clarity and additional analysis, but did not receive any.	Reported statistics were used.
Black WHS, US[35]	SSB and sugar-sweetened berry juices were reported separately. Results after adjustment for body-mass index were presented partially (estimates for the extreme categories) and presented by stratification. We requested the authors to do analysis combining the two beverage types, but could not obtain any information.	Reported statistics for SSB were used.
MESA, US[36]	The author confirmed no availability of additional information. ‡	The article reported null associations between SSB consumption and incidence of T2D, but available in a review article.[37]
Occupational cohort, Japan[38]	We identified availability of fruit juice based on a publication on dietary assessment they used. Thus, we requested estimates for fruit juice consumption and incident T2D, as well as for SSB and ASB, with and without adjustment for adiposity measures. The author responded to our request.	Estimates for SSB, ASB, and fruit juice consumption were used, as provided. The update was unlikely to involve any bias.

Abbreviations: ARIC, Atherosclerosis Risk in Communities Study; ASB, Artificially-sweetened beverages; BRHS, British Regional Heart Study; CARDIA, Coronary Artery Risk Development in Young Adults Study; D.E.S.I.R., Data from an Epidemiological Study on the Insulin Resistance Syndrome; EBSHP, The East Boston Senior Health Project; EPIC,

European Prospective Investigation into Cancer and Nutrition Study; EPIC-NL, EPIC-Netherlands Study; FMCHES, Finnish Mobile Clinic Health Examination Survey; FOS, Framingham Offspring Study (Framingham Heart Study, the second generation); HIPOP-OHP, the High-risk and Population Strategy for Occupational Health Promotion Study; HPFS, Health Professional Follow-up Study; JPHC, Japan Public Health Center-based Prospective Study; KIHDS, Kuopio Ischaemic Heart Disease Risk Factor Study; MESA, Multi-Ethnic Study of Atherosclerosis; NHS, Nurses' Health Study; NIH-AARP, National Institute of Health American Association of Retired Persons Diet and Health Study; OGTT, oral-glucose tolerance test; PHS, Physicians Health Study; SCHS, Singapore Chinese Health Study; SSB, sugar-sweetened beverages; SUN, Sequimiento University of Navarra; T2D, type 2 diabetes; WHS, Women's Health Study.

* When we contacted the authors (October-December, 2013), we specified our requests to obtain categorical and continuous estimates before and after adjustment for obesity status for prospective associations between each type of SSB, ASB, and fruit juice and incident T2D. We specified statistical methods, categorization, and covariates adjusted for, based on prior publications. After we obtained information usable in this meta-analysis, we did not request further information.

† Some exclusion might be related to publication bias, because these cohorts could technically provide information useful for this meta-analysis; although technically available, According to the publications not included in this meta-analysis, 283,058 of whom 23,270 cases arose were not included in this meta-analysis in total.

‡ The authors reported estimates after stratification by demographics or by body-mass index, we merged the estimates by fixed-effect meta-analysis in main analysis. In analyses to test heterogeneity by demographics or bod0mass index, stratified results were

Table S2. Quality assessment of cohort studies included in meta-analysis of sweet beverages and incidence of type 2 diabetes.

Cohort	Domains of bias*, + Low risk of bias, − High risk of bias, ? unknown.								Additional consideration on potential sources of bias
	Con-founding	Selection	Dietary measures	Follow-up	Missing data	Diagnosis of T2D	Selective report	Overall †	
NHS I[14,15]	?	+	?	+	+	?	+	?	Analyses were updated. A risk of bias was unlikely to be high.
NHS II [14,18,19]	?	+	?	+	+	?	+	?	Analyses were updated. A risk of bias was unlikely to be high.
ARIC[25]	?	+	−	−	+	+	−	−	SSB and ASB were not separated.
Iowa WHS[23]	?	?	−	−	?	?	−	−	Only the conference abstract was published.
FOS[26]	?	+	−	+	+	+	?	?	Modified substantially for updating the original analysis.
HPFS[39,40]	?	+	?	+	+	?	+	?	Analyses were updated. A risk of bias was unlikely to be high.
Black WHS[41]	?	+	?	+	+	?	−	?	Results were reported selectively.
MESA[36]	?	+	−	−	+	+	−	?	Results were reported selectively.
EPIC-InterAct[31]	?	+	?	−	+	?	+	?	A risk of bias was unlikely to be high.
E3N[42]	−	+	?	−	+	?	+	−	Adjustment for adiposity was likely to be biased.[43–46]
SCHS[47]	?	+	−	−	−	?	?	−	Fruit juice and fruit drinks (SSB) were not separated. Exclusion might have caused bias.
JPHC[48]	?	+	?	−	+	?	−	?	Main and subgroup analyses were internally and externally inconsistent.[28,30]
Occup. cohort[49]	?	+	−	−	+	+	?	?	Modified substantially for updating the original analysis.
HIPOP-OHP[50]	?	+	−	−	−	+	?	−	Exclusion might have caused bias, losing 31% of participants during the follow-up.
CARDIA[51]	?	+	−	+	?	−	−	−	Main and subgroup analyses were internally and externally inconsistent.[21,22]
KIHDS[52]	?	+	−	−	+	+	+	?	Habitual consumption not measured well.
FMCHES[53,54]	?	+	?	−	+	+	+	?	Generalizability to the modern population is concerning.

Abbreviations: ARIC, Atherosclerosis Risk in Communities Study; CARDIA, Coronary Artery Risk Development in Young Adults Study; EPIC, European Prospective Investigations into Cancer and Nutrition Study; FMCHES, Finnish Mobile Clinic Health Examination Survey; FOS, Framingham Offspring Study (Framingham Heart Study, the second generation); HIPOP-OHP, High-risk and Population Strategy for Occupational Health Promotion Study; HPFS, Health Professional Follow-up Study; JPHC, Japan Public Health Center-based Prospective Study; KIHDS, Kuopio Ischaemic Heart Disease Risk Factor Study; MESA, Multi-Ethnic Study of Atherosclerosis; NHS, Nurses' Health Study; SCHS, Singapore Chinese Health Study; SSB, WHS, Women's Health Study.

* See supplementary text for details. Influence of sources of bias were examined in sensitivity analyses (Table S5 and S6). For dietary measures, bias was considered as high, if quality of dietary measures was not assessed within a study; as unknown for the other studies, with possible misclassification. Follow-up was qualified by availability of repeated dietary measures. Missing data on exposure were considered unlikely to cause bias in any studies. SCHS and HIPOP-OHP lost 15% and 31% of participants, respectively, in follow-up. Bias for type 2 diagnosis (T2D) diagnosis was assessed as a low risk, if a study took approach to detect undiagnosed diabetes. See also Table S3 for validity measures of dietary measures and ascertainment of T2D; and Table S5, for potential confounders.

† Overall bias reflects possibility of bias specifically on the estimates used in the meta-analysis (see the first right column and the supplementary text on page 16). Sensitivity meta-analysis was performed after excluding these studies (Table S6).

Table S3. Validity measures of beverage consumption and incident type 2 diabetes.

Cohort*	Internal substudy †	Reference ×n of assessments	N	SSB r‡	SSB s_Q/s‡	ASB r‡	ASB s_Q/s‡	Fruit juice r‡	Fruit juice s_Q/s‡	Self-report only	N cases identified (Person-years, ×1000)	PPV, n for validation
	Assessment of within-person variability of dietary estimates by FFQ or DR									Ascertainment of incident type 2 diabetes§		
NHS I[55,56]	Yes	7d DR ×4	173	0.84	1.83	0.36	1.83	0.84	1.00	X	7,449 (1,571)	0.98, 62
NHS II	No									X	5,225 (1,660)	0.98, 62
ARIC[25]	No										1,437 (92.5)	
Iowa WHS[24]	No									X	1,561 (330.0)	0.64, 44
FOS[27,57]	No										303 (33.3)	
HPFS[39,40,58]	Yes	7d DR ×2	127	0.84	2.37	0.40	2.24	0.82	1.66	X	3,364 (777.3)	
Black WHS[35,41]	Yes	7d DR ×4	403	0.67	1.17	0.67	1.17	0.64	1.19	X	2,713 (338.9)	0.94,229
MESA[36,59]	No	7d DR ×4	186	0.46	0.71	0.46	0.71	‖	‖		413 (27.6)	
EPIC-InterAct[33] **	Yes	24hR ×12, 24, or 10, 4d DR ×4, or 7d DR ×2	999	0.65	1.13	0.64	1.14	0.73	1.30		11,684 (3,990)	
E3N[32,42]	Yes	24hR × 9-12	119	0.55	1.22	0.55	1.22	0.55	1.22		1,054 (607.0)	
SCHS[47,60]	Yes	24hR ×2	810	0.49	1.20	‖	‖	0.58	1.29		2,273 (249.2)	0.99, 702
JPHC (men) [48,61]	Yes	7d DR ×4 or 2	94	0.27	2.46	‖	‖	0.17	2.46	X	824 (271.7)	0.82, 93
(women)	Yes	7d DR ×4 or 2	107	0.24	2.46	‖	‖	0.18	2.46			
Occup. cohort, Japan[38,49]	No	7d DR ×4	92	0.39	1.06	0.39	1.06	0.24	1.98		170 (11.3)	
HIPOP-OHP[12,50]	Yes	24hR ×4	76	0.32	2.00	‖	‖	0.32	2.00		212 (20.8)	
CARDIA[21,51,62]	Yes	24hR ×7	128	0.68	1.90	0.68	1.90	0.59	1.78		288 (67.2††)	0.62
KIHDS[20,52,63]	No	24hR ×10	96	0.68	1.00	‖	‖	‖	‖		506 (46.8)	
FMCHES[13,53,54]	No	7d DR ×1	79	0.62	1.17	‖	‖	‖	‖		175 (58.8)	

Abbreviations: 24hR, 24-hour recalls; ARIC, Atherosclerosis Risk in Communities Study; ASB, artificially-sweetened beverages; CARDIA, Coronary Artery Risk Development in Young Adults Study; DR, diet records; EPIC, European Prospective Investigations into Cancer and Nutrition Study; FFQ, food-frequency questionnaires, FMCHES, Finnish Mobile Clinic Health Examination Survey; FOS, Framingham Offspring Study (Framingham Heart Study, the second generation); HIPOP-OHP, High-risk and Population Strategy for Occupational Health Promotion Study; HPFS, Health Professional Follow-up Study; JPHC, Japan Public Health Center-based Prospective Study; KIHDS, Kuopio Ischaemic Heart Disease Risk Factor Study; MESA, Multi-Ethnic Study of Atherosclerosis; NHS, Nurses' Health Study; PPV; predictive positive value; SCHS, Singapore Chinese Health Study; SSB, sugar-sweetened beverages; WHS, Women's Health Study.

* Citations represent articles we cited to derive measures of within-person variability of assessment of consumption of SSB, ASB, and fruit juice; and of validity of case ascertainment.

† NHS II, ARIC and Iowa WHS used the questionnaires developed for the nurses in the NHS I. ARIC aggregated SSB, ASB, and fruit juice in their analysis, and correlations were averaged after Z-transform. FOS, FFQs for the NHS I at the analysis baseline and for the HPFS at the follow-up; MESA, FFQ developed for multi-ethnic populations in the Insulin Resistance Atherosclerosis Study; the occupational cohort in Japan, FFQ developed and validated in another setting. Iowa WHS reported internal validation study was published[64], but we did not use it, because reference methods (24hR × 5) were implemented only in February and March, and we considered the study was logistically unable to validate the FFQ designed to capture 1-year habitual diet. Finish cohorts did not perform internal validation studies to examine whether each method could capture habitual dietary

consumption. Thus, two studies in Canada and in Netherlands validating a diet-history method were reviewed. Because of similarity in geography, the study in Netherlands was focused; yet, validity for assessment of sugar intake was similar (r=0.62 and 0.60) in the two studies.

‡ r represents correlation coefficients between estimates based on FFQ or diet history and estimates based on average of reference methods. We used energy-adjusted estimates corrected for within-person variations, if available.[65,66] s_Q/s represents a ratio of a standard deviation of FFQ or diet history to that of a reference method; for diet records, standard deviations were assumed to be unbiased. If specific measures of r and s_Q/s were not available for SSB, ASB, or fruit juice, variables related to refined sugars (disaccharides, sucrose, or carbohydrates) were used for SSB and ASB, and averages of variables related to sugars and vitamin C intakes were used for fruit juice. Averages of correlations were based on Z-transformed values[67,68]; of ratios, log-transformed values.

§ For studies using objective measures of diagnosis (Table 1), PPV was assumed to be 1.0.Person-year was coded as presented or imputed by using the number of participants, the number of incident cases, and the maximum duration of follow-up. The presented numbers of cases and person-years were not corrected for positive predictive values (PPV). Thus, some values were different from those in Table 1.

|| Beverages were not assessed for associations with incident diabetes and not included in this meta-analysis.

** In EPIC-InterAct, FFQs were developed specifically in each of the eight countries of the consortium. Measures of validity and reliability were calculated by weighted averages of the measures from the eight cohorts[42,52,69–78] (available on request), for which weights were those of country-specific estimates to the overall estimates in EPIC-InterAct. The total number of adults were based on the number of adults contributing to the measures of validity for SSB and ASB. For fruit juice, N was 1,258.

†† CARDIA reported associations of beverage consumption with hyperglycaemia. We included the study in this meta-analyses, considering the overlapping definitions of hyperglycaemia and incident type 2 diabetes (use of antidiabetic medications). PPV represents the proportion of patients with type 2 diabetes to patients with hyperglycaemia.

Table S4. Assessment of potential confounders in the studies included in the meta-analysis of beverage consumption and type 2 diabetes.

Cohort	Potential confounders accounted by sampling or statistical adjustment								Relative risk (95% confidence interval)					
	Age, sex, race*	SES *	Smoke	Physical activity	Alcohol	Diet †	Clinical factors ‡	SSB, ASB, fruit juice §	SSB Crude	SSB Adjusted ‖	ASB Crude	ASB Adjusted ‖	Fruit juice Crude	Fruit juice Adjusted ‖
FMCHES[13]	✓	✓	✓		✓	✓	✓		1.94	na ‖	na	na	na	na
NHS I[14,15]	✓	✓	✓	✓	✓	✓	✓	✓	1.51	1.39 (1.30-1.48)	1.42	1.24 (1.19-1.30)	1.42	1.24 (1.19-1.30)
NHS II[14,18,19]	✓	✓	✓	✓	✓	✓	✓	✓	1.31	1.17 (1.11-1.24)	1.36	1.20 (1.16-1.25)	1.36	1.20 (1.16-1.25)
HPFS[14,16,17]	✓	✓	✓	✓	✓	✓	✓	✓	1.21	1.31 (1.20-1.44)	1.51	1.23 (1.15-1.32)	1.51	1.23 (1.15-1.32)
KIHDS[20,63]	✓	✓	✓	✓	✓	✓	✓		0.97	1.06 (0.95-1.18)	na	na	na	na
CARDIA[21,22]	✓		✓	✓	✓	✓	✓	✓	na	na	na	na	na	na
Iowa WHS[23]	✓	✓	✓	✓	✓	✓	✓		na	na	na	na	na	na
FOS[26,27]	✓		✓	✓	✓	✓	✓	✓	1.25	1.12 (0.90-1.40)	1.35	1.24 (1.13-1.37)	1.35	1.24 (1.13-1.37)
ARIC[25]	✓	✓	✓	✓	✓	†	✓		1.08	1.01 (0.96-1.06)	na	na	na	na
JPHC[28]	✓	✓	✓	✓	✓	✓	✓		1.21	1.25 (0.99-1.58)	na	na	na	na
EPIC-InterAct[31]	✓	✓	✓	✓	✓	†	✓	✓	1.39	1.21 (1.12-1.31)	1.60	1.36 (1.18-1.56)	1.60	1.36 (1.18-1.56)
E3N[32]	✓	✓	✓	✓	✓	✓	✓		2.64	2.82 (0.87-9.17)	12.6	11.7 (4.03-34.3)	12.6	11.7 (4.03-34.3)
SCHS[34]	✓	✓	✓	✓	✓	✓		✓	2.04	2.22 (1.64-3.00)	na	na	na	na
Black WHS	✓	✓		✓	✓	✓	✓	✓	1.16	1.10 (1.05-1.16)	na	1.05 (0.86-1.27)	na	1.05 (0.86-1.27)
HIPOP-OHP[12]	✓	✓	✓	✓	✓	✓	✓		0.79	0.89 (0.75-1.06)	na	na	na	na
MESA[36]	✓	✓	✓	✓	†	†		✓	na	na	1.35	1.48 (1.21-1.80)	1.35	1.48 (1.21-1.80)
Occup. Japan[38]	✓	✓	✓	✓	✓	✓	✓	✓	1.12	1.08 (0.88-1.33)	3.17	1.34 (0.90-1.99)	3.17	1.34 (0.90-1.99)
Pooled (Table 2)									1.25	1.18 (1.09-1.28)	1.48	1.25 (1.18-1.33)	0.97	1.05 (0.99-1.11)

Abbreviations: ARIC, Atherosclerosis Risk in Communities Study; ASB, artificially sweetened beverages; CARDIA, Coronary Artery Risk Development in Young Adults Study; EPIC, European Prospective Investigation into Cancer and Nutrition Study; FMCHES, Finnish Mobile Clinic Health Examination Survey; FOS, Framingham Offspring Study (Framingham Heart Study, the second generation); HIPOP-OHP, High-risk and Population Strategy for Occupational Health Promotion Study; HPFS, Health Professional Follow-up Study; JPHC, Japan Public Health Center-based Prospective Study; KIHDS, Kuopio Ischaemic Heart Disease Risk Factor Study; MESA, Multi-Ethnic Study of Atherosclerosis; NHS, Nurses' Health Study; PA, physical activity; SCHS, Singapore Chinese Health Study; SSB, sugar sweetened beverages; WHS, Women's Health Study.

* Age was considered adjusted for in a cohort using it as a time-scale in longitudinal analysis (NHS I, NHS II, HPFS, EPIC-InterAct, and E3N). Race and socioeconomic status (SES) were considered as adjusted for in some cohorts recruiting participants in a population homogenous in race/ethnic status and in occupation (FMCHES, NHS I, NHS II, HPFS, E3N, HIPOP-OHP, and occupational cohort in Japan). Another case of adjustment for SES was inclusion of education history in a multivariable regression analysis. Black WHS included a job status as a covariate, in addition to education history.

† Dietary factors were not adjusted in main analyses in EPIC-InterAct and MESA. ARIC did dietary adjustment for intakes of alcohol, total calorie, and fibre only. EPIC-InterAct and MESA confirmed little influence of potential dietary confounders in secondary analyses. The lack of substantial influence was also confirmed in NHS I, NHS II, and HPFS.

‡ Clinical factors mean either family history of diabetes, use of anti-hypertensive or lipids-lowering drugs, or history of cardiovascular diseases, hypercholesterolemia, or hypertension. Family history of diabetes was adjusted for in FMCHES, NHS I, NHS II, KIHDS, HPFS, Iowa WHS, ARIC, JPHC, E3N, Black WHS, HIPOP-OHP, occupational cohort in Japan. EPIC-InterAct did not collect family history of diabetes among 51.7% of the random sub-cohort, and not used it in the main analysis, but sensitivity analysis excluding adults with known family history of diabetes confirmed little influence of the variable.[31]

§ Checked if different types of beverages were mutually adjusted for. NHS I, NHS II, and HPFS confirmed that mutual adjustment did not affect results.

‖ Relative risk (95% confidence interval) adjusted for potential confounders except adiposity measures. Models adjusted for adiposity measures are presented in Table 2 and Figure 1. 'na' indicates that the authors did not report statistics for the specific estimate. For example, CARDIA and IowaWHS reported adiposity-adjusted estimates only.

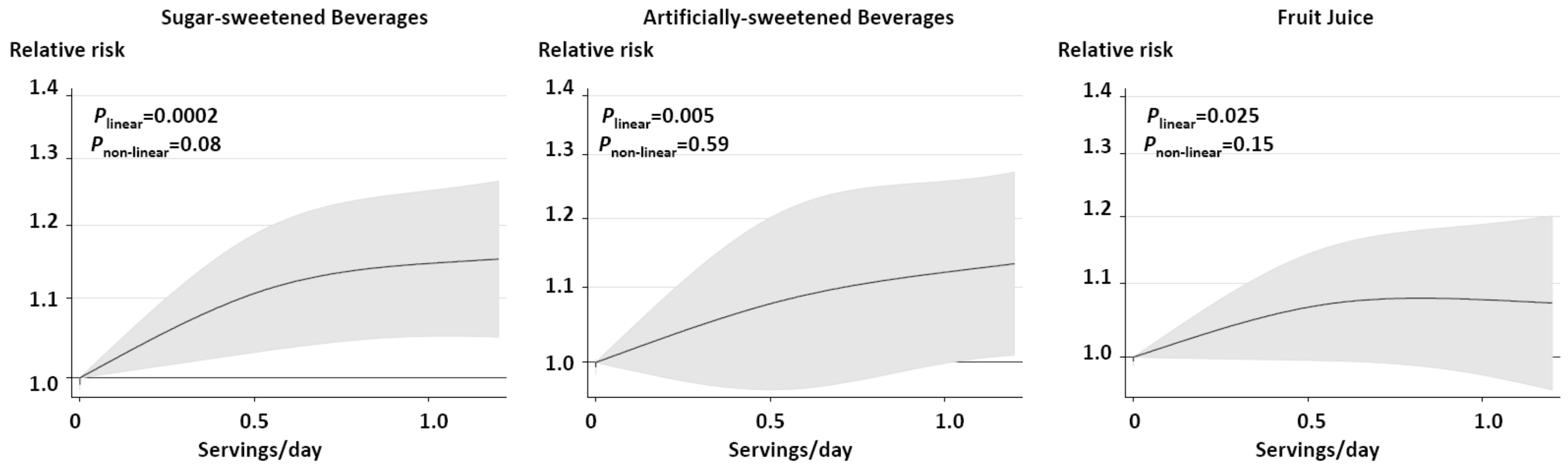

Figure S2. Non-linear associations of consuming sugar-sweetened beverages, artificially-sweetened beverages, and fruit juice with incident type 2 diabetes. Estimates were obtained by random-effects meta-analysis adjusted for adiposity. The curves and *P* for a non-linear associations ($P_{non\text{-}linear}$) were obtained by cubic spline meta-analysis.[79] Solid lines are the central estimates of relative risks (RR) and shaded areas are the corresponding 95% confidence interval (CI). The analysis needed categorical estimates, rather than continuous estimates per one serving/day, thus we needed to drop the studies reporting only continuous estimates: for sugar-sweetened beverages, 13 estimates were used, not including European Prospective Investigations into Cancer and Nutrition Study (EPIC)-InterAct and Coronary Artery Risk Development in Young Adults Study (CARDIA), Iowa Women's Health Study and Multiethnic Study of Atherosclerosis; for artificially-sweetened beverages, 7 estimates were used, not including EPIC-InterAct, CARDIA; for fruit juice, 10 estimates were used, not including EPIC-InterAct, CARDIA. *P* for a linear association (P_{linear}) was obtained by meta-analysis using all estimates available. Using the limited categorical data, calibration for within-individual variability applied to categorical estimates[80,81] provided steeper effects with similar curves and wide CI (data not shown).

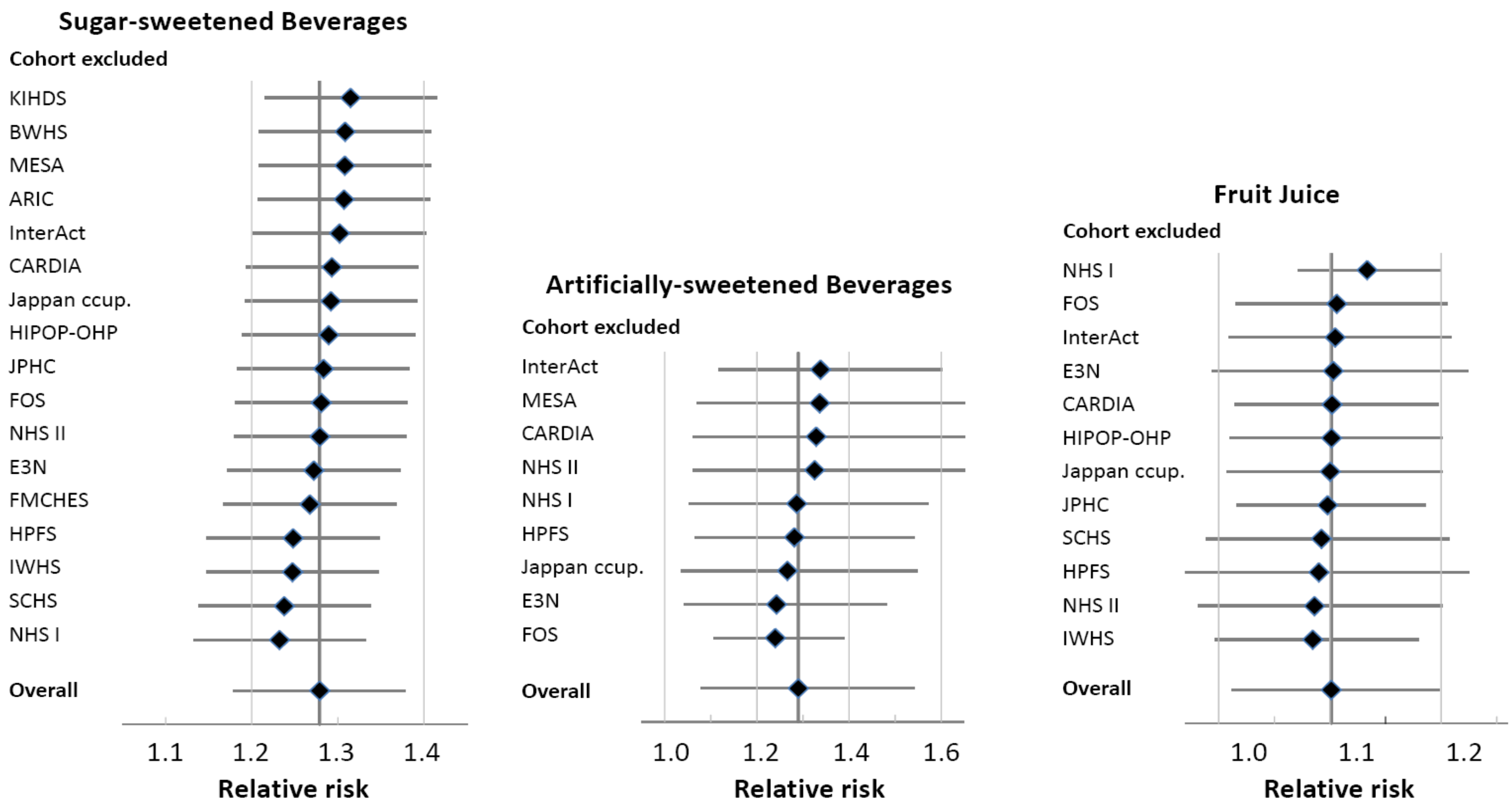

Figure S3. Influence analysis for the prospective associations of consuming sugar-sweetened beverages, artificially-sweetened beverages, and fruit juice with incident type 2 diabetes.

All estimates were obtained by random-effects meta-analysis adjusted for adiposity and for within-person variability of beverage consumption and precision of diabetes diagnosis. Overall estimates were based on analysis using all estimates from the studies presented for each beverage. The estimate accompanied to each cohort was based on meta-analysis excluding the study. Variations in relative risks ranged from -19% to +16% for SSB, -20% to +23% for ASB, and -7% to +16% for fruit juice. Abbreviations: ARIC, Atherosclerosis Risk in Communities Study; CARDIA, Coronary Artery Risk Development in Young Adults Study; FMCHES, Finnish Mobile Clinic Health Examination Survey; FOS, Framingham Offspring Study (Framingham Heart Study, the second generation); HIPOP-OHP, High-risk and Population Strategy for Occupational Health Promotion Study; HPFS, Health Professional Follow-up Study; JPHC, Japan Public Health Center-based Prospective Study; KIHDS, Kuopio Ischaemic Heart Disease Risk Factor Study; MESA, Multi-Ethnic Study of Atherosclerosis; NHS, Nurses' Health Study; SCHS, Singapore Chinese Health Study; SSB, sugar-sweetened beverages; WHS, Women's Health Study.

Table S5. Potential sources of heterogeneity for the prospective associations of consuming sweet beverages with incident type 2 diabetes.

Potential sources of heterogeneity	Sugar-sweetened beverages		Artificially-sweetened beverages		Fruit juice	
(n cohorts)*	RR (95% CI) †	$P_{heterogeneity}$‡	RR (95% CI) †	$P_{heterogeneity}$‡	RR (95% CI) †	$P_{heterogeneity}$‡
**Geographic location**						
United States (n=9)	1.22 (1.07-1.38)		1.33 (1.06-1.67)		1.10 (0.99-1.22)	
Europe (n=4)	1.53 (1.12-2.09)		1.50 (0.98-2.30)		1.05 (0.90-1.21)	
Singapore or Japan (n=4)	0.94 (0.43-2.08)	0.39	1.83 (0.59-5.68)	0.92	0.42 (0.02-7.19)	0.51
**Age on average**						
<53 years (n=9)	1.20 (1.07-1.33)		1.13 (0.89-1.44)		1.15 (0.99-1.33)	
≥53 years (n=8)	1.36 (1.10-1.68)	0.16	1.49 (1.19-1.87)	0.12	1.08 (0.97-1.19)	0.48
**Sex, proportion>50%**						
Women (n=10)	1.28 (1.13-1.46)		1.42 (1.14-1.77)		1.08 (0.98-1.18)	
Men (n=7)	1.17 (0.90-1.53)	0.42	1.28 (0.92-1.78)	0.59	1.14 (0.95-1.36)	0.88
**Body-mass index on average†**						
<26.0 kg/m^2 (n=8)	1.45 (1.16-1.81)		1.41 (1.03-1.92)		1.11 (1.04-1.19)	
≥26.0 kg/m^2 (n=9)	1.16 (1.03-1.30)	0.49	1.38 (1.07-1.79)	0.41	1.08 (0.93-1.26)	0.57
**Incidence of type 2 diabetes**						
<6.0 / 1,000 person-years (n=8)	1.53 (1.27-1.85)		1.41 (1.09-1.81)		1.13 (1.07-1.20)	
≥6.0 / 1,000 person-years (n=9)	1.12 (1.00-1.25)	0.12	1.37 (1.00-1.87)	0.34	1.01 (0.85-1.20)	0.48
**Duration of follow-up**						
<10 years (n=6)	1.11 (0.93-1.32)		1.70 (1.23-2.36)		1.15 (0.14-9.35)	
≥10 years (n=11)	1.36 (1.19-1.56)	0.68	1.33 (1.07-1.64)	0.13	1.09 (1.02-1.17)	0.96
**N of dietary measurements**						
Once, only at baseline (n=11)	1.28 (1.06-1.56)		1.55 (1.19-2.01)		1.10 (0.97-1.24)	
Repeated (n=6)	1.26 (1.11-1.43)	0.25	1.24 (0.96-1.61)	0.90	1.08 (0.97-1.20)	0.068
**Ascertainment of type 2 diabetes**						
Self-reported only (n=6)	1.36 (1.15-1.60)		1.22 (1.05-1.42)		1.15 (1.08-1.22)	
Objective measures (n=11)	1.19 (1.02-1.39)	0.20	1.52 (1.14-2.03)	0.93	0.98 (0.86-1.11)	0.008

* Stratified analysis was prespecified for demographics and factors significantly predicting heterogeneity of associations for any type of beverages (p<0.1). For each type of beverages, a fewer cohorts contributed to the estimates: sugar-sweetened beverages, n=17 in total; artificially sweetened beverages, n=9; and fruit juice, n=12.

† Random-effects meta-analysis was performed in each stratum to estimate relative risks (RR) and 95% confidence intervals (CI). All estimates were adjusted for within-person variations and precision of type 2 diabetes diagnosis. If cohorts reported estimates after stratification by demographics and after adjustment for adiposity measures, the stratified estimates were used, for example, estimates stratified by sex in the Atherosclerosis Risk in Communities Study and in Japan Public Health Center-based Prospective Study. Use of stratified estimates had more precise estimates. For example, when restricting populations to those with BMI<26.0 kg/m^2, RRs for sugar-sweetened beverages were 1.45 (1.16-1.81) with stratified estimates and 1.52 (1.11-2.06) without stratified estimates; and BMI≥26.0 kg/m^2, 1.16 (1.03-1.30) with stratified estimates and 1.17 (1.03-1.33) without stratified estimates.

‡ *P* for heterogeneity. Significant (*P*<0.1) for repeated measures of dietary assessments and ascertainment of type 2 diabetes (the last two sets of rows) in the analysis of fruit juice. Variables with *P*<0.2 were mutually adjusted. Variables with *P*>0.2 were obtained in the model including the variables meeting the criterion of P<0.2 for entry. Heterogeneity was not significant (*P*>0.1) for the other factors for any types of beverages: duration of follow-up, use of FFQ or other methods (Table S3), selective reporting (yes or no, Table S2), publication status (peer-reviewed or not), and mutual adjustment for three different beverages (yes or no, Table S4).

Table S6. Associations of consuming sweet beverages with incident type 2 diabetes by sensitivity meta-analysis.

Consideration (n estimates)	Sugar-sweetened beverages		Artificially-sweetened beverages		Fruit juice	
	N studies	RR (95% CI)*	N studies	RR (95% CI)*	N studies	RR (95% CI)*
**Random-effects or fixed-effects modelling.**						
Random-effects.	17	1.28 (1.12-1.46)	9	1.29 (1.08-1.54)	12	1.10 (1.01-1.20)
Fixed-effects.	17	1.21 (1.14-1.28)	9	1.23 (1.06-1.44)	12	1.11 (1.05-1.17)
**Unit of beverage consumption.**						
per 1 serving/day (original estimates).	17	1.28 (1.12-1.46)	9	1.29 (1.08-1.54)	12	1.10 (1.01-1.20)
per 250 ml/day. †	17	1.28 (1.11-1.47)	9	1.25 (1.06-1.48)	12	1.13 (1.01-1.25)
**Selected studies**						
Studies without possibility of overall bias. ‡	11	1.25 (1.10-1.41)	7	1.26 (1.13-1.41)	8	1.09 (1.00-1.19)
Studies without less valid dietary assessment. §	16	1.30 (1.13-1.49)	6	1.28 (0.92-1.80)	9	1.10 (1.01-1.20)
Studies verifying internal validity of dietary assessment‖	8	1.28 (1.19-1.37)	5	1.21 (1.07-1.37)	6	1.13 (1.06-1.19)
**Aggregation of cohorts in the consortium analysis.‖**						
Cohorts within EPIC-InterAct, aggregated.	17	1.43 (1.20-1.70)	9	2.13 (1.57-2.88)	12	1.06 (0.98-1.14)
Cohorts within EPIC-InterAct, separated.	25	1.40 (1.22-1.61)	17	2.00 (1.57-2.54)	19	1.06 (1.00-1.14)
**Analysis accounting for errors of measures of validity**						
Estimates after accounting for precision of ln(RR), measures of validity of exposure (γ), and PPV ††	17 ×10,000	1.29 (1.10-1.53)	9 ×10,000	1.33 (1.06-1.11)	11 ×10,000	1.11 (1.00-1.25)
**Calibrated for potential misclassification for adiposity measurements. ‡‡**						
r_a = 0.9 between observed and true adiposity measures	17	1.22 (1.07-1.41)	9	1.08 (0.87-1.34)	11	1.12 (1.02-1.21)
r_a = 0.8	17	1.20 (1.04-1.38)	9	1.01 (0.81-1.25)	11	1.12 (1.03-1.22)
r_a = 0.7	17	1.17 (1.02-1.35)	9	0.93 (0.75-1.15)	11	1.13 (1.04-1.23)

Abbreviations: CI, confidence interval; EPIC, European Prospective Investigation into Cancer and Nutrition Study; PPV, positive predictive value; RR, relative risk.

* Random effects meta-analysis was performed in each stratum, except for the estimates derived from fixed-effects modelling. All were adjusted for adiposity measures and calibrated for misclassification of exposure and outcome.

† Median serving size of beverage consumption in the cohorts included in this present meta-analysis. Different studies defined one serving differently.

‡ Bias was determined by qualitative assessment (Table S2).

§ defined as r<0.4 compared to reference methods). Relatively low validity for dietary assessment (Table S3) was also used as a source of bias. As the validity measures were not all specific to each beverage, the results were interpreted cautiously as supplements.

‖ defined as studies that conducted internal validation study to confirm if a dietary assessment was likely to capture habitual diet in a study population (Table S2)

** Using all cohorts available, but EPIC-InterAct was considered as a single cohort or separated.[31] The publication did not report the cohort-specific estimates adjusted for measures of adiposity. Thus, in the main analyses, we used the estimates combined within EPIC-InterAct. Additionally, the publication reported the cohort-specific estimates (11 cohorts in total from 8 countries) without adjustment for measures of adiposity.[31] Thus, the sensitivity to the aggregation was assessed here. Cohort-specific calibration for dietary measurement errors was applied.

†† Iterative sensitivity analysis (10,000 times) was performed after incorporating quantitative bias and uncertainty[82] in different measures: dose-response estimates, within-person variability of beverage consumption, and precision of incident diabetes. Uncertainty of each was randomly drawn from each standard error. Out of 10,000 repeats, 2.5th, 50th (median), and 97.5th percentiles were obtained for 95% confidence limits and point estimate of RR.

‡‡ Estimates were obtained after adopting specific unobserved, but realistic assumptions: 1) adiposity was measured with misclassification (r_a); 2) observed estimates adjusted for measured adiposity were biased to the extent related to r_a; and 3) estimates calibrated for r_a were obtained by a formula following simulation extrapolation (see text and Figure S4). A recent article[83] indicated r_a was greater than 0.73, thus assumed to be 0.7 or higher.

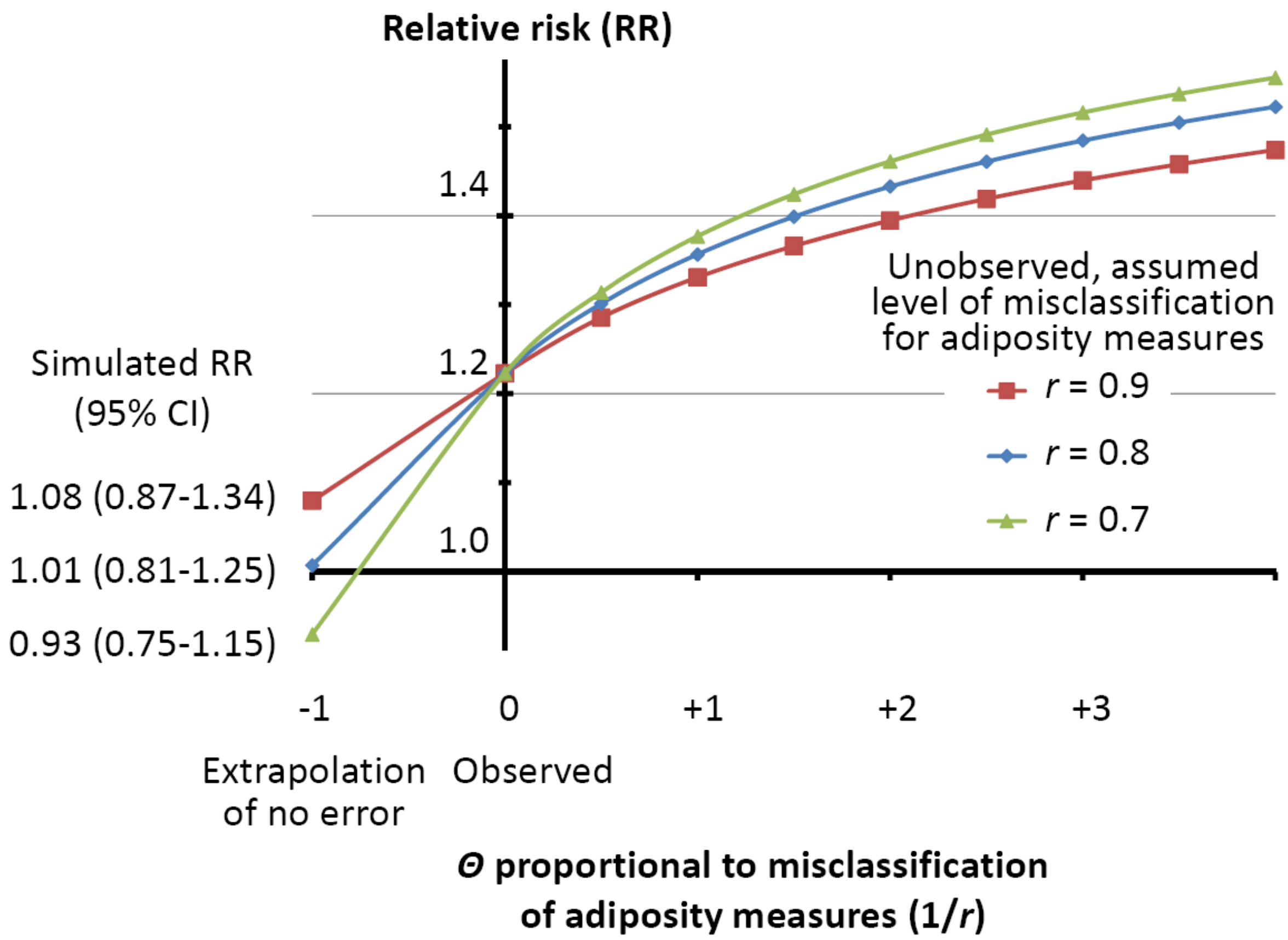

Figure S4. Assessment of prospective association of consuming artificially sweetened beverages with incident type 2 diabetes after adjustment for assumed misclassification of adiposity measurements.

Estimates were obtained after adopting specific unobserved, but realistic assumptions: 1) adiposity was measured with misclassification (r_a); 2) observed estimates adjusted for measured adiposity were biased to the extent related to r_a; and 3) estimates calibrated for r_a were obtained by a formula following simulation extrapolation, $\frac{\sigma_{between}}{\sigma_{between}+\sigma_{within}(1+\theta)}$; θ=0 would produce observed RR=1.20 (0.98-1.52); $\theta=\infty$ would produce ln(RR) unadjusted for adiposity measure, RR=1.80 (1.13-2.46); θ=-1 would produce ln(RR) adjustged for potential misclassification of measured adiposity. The extrapolation for θ=-1 from the observable range, θ>0, was performed by non-linear association derived from θ ={0, 0.5, 1.0, 1.5, 2.0} of which functionality was demonstrated for another analysis.[84] A recent article[83] indicated r_a was greater than 0.73, thus we assumed r_a to be 0.7 or higher.

Table S7. The number of type 2 diabetes events preventable over 10 years from 2010 by eliminating sugar-sweetened beverage consumption among adults in the United States and in the United Kingdom.*

Population	Adults free of diabetes, N / 1,000	Consumption of SSB, mean±SD, g/day (% consumers)†	T2D in 10 years, N / 1,000 (10-year risk) ‡	T2D events prevented by eliminating SSB consumption §			
				Unadjusted for adiposity		Adjusted for adiposity	
				N / 1,000	PAF (95% CI)	N / 1,000	PAF (95% CI)
**United States**							
All	189,076	284±412 (54.4)	20,878 (11.0)	2,564	11.9 (7.4-16.5)	1,824	8.7 (3.9-12.9)
Age, years							
20-44	97,586	384±435 (65.3)	7,317 (7.5)	1,556	20.2 (12.0-28.3)	1,102	15.1 (13.6-16.5)
45-64	64,940	204±294 (44.8)	9,179 (14.1)	800	8.5 (4.7-12.4)	572	6.2 (5.4-7.1)
≥65	26,550	109±228 (37.7)	4,381 (16.5)	208	4.7 (2.7-6.7)	150	3.4 (3.0-3.9)
Sex							
Men	89,692	373±457 (61.7)	9,948 (11.1)	1,626	15.7 (9.2-22.2)	1,152	11.6 (10.7-12.5)
Women	99,383	203±295 (47.8)	10,930 (11.0)	937	8.4 (4.7-12.2)	673	6.2 (5.4-6.9)
**United Kingdom**							
All	44,719	114±157 (49.4)	2,593 (5.8)	126	4.9 (3.0-7.2)	79	3.6 (1.7-5.6)
Age, years							
20-44	20,865	166±181 (63.0)	441 (2.1)	38	8.6 (5.5-12.8)	21	6.5 (2.9-10.0)
45-64	14,937	78±126 (39.9)	1,195 (8.0)	59	4.9 (3.1-7.4)	37	3.7 (1.7-5.6)
≥65	8,920	53±95 (33.6)	954 (10.7)	29	3.1 (1.9-4.7)	21	2.4 (1.0-3.7)
Sex							
Men	21,243	135±173 (51.6)	1,170 (5.5)	67	5.8 (3.4-8.1)	43	4.3 (1.8-6.4)
Women	23,474	95±140 (47.4)	1,423 (6.1)	59	4.1 (2.5-6.0)	36	3.1 (1.4-4.7)

* ×1, 000 for counts (N) derived from the United States National Health and Nutrition Examination Survey, 2009-2010 (n=4,729 adults free of diabetes) and from the United Kingdom National Dietary Nutrition Survey, 2008/2009-2011/2012 (n=1,932 adults free of diabetes) (Supplementary Methods for details). All statistics accounted for sampling weights. PAF, population attributable fraction; SD, standard deviation, SSB, sugar-sweetened beverages; T2D, type 2 diabetes.

† The distribution of SSB consumption was positively skewed in every population group. In data from the United States, consumers were defined by consumption of sugar-sweetened beverage at least once in a 24-hour recall of dietary consumption or by daily consumption reported in a dietary screener questionnaire. In those from the United Kingdom, consumers were defined as adults who recorded consumption of any of SSB during four days of dietary recording.

‡ 10-year risk of T2D was predicted using measured risk factors for T2D and a published risk-prediction algorithm in each of the United States and the United Kingdom.

§ Calculated based on the difference between the predicted T2D risk varying according to observed SSB consumption and the counterfactual T2D risk if no one in each population consumes SSB. The risks associated with SSB were estimated under different assumptions: Left. the effect of SSB consumption was partly mediated by obesity, modelled with relative risk unadjusted for adiposity measures; and Right. the effect of SSB was independent of obesity, modelled with relative risk adjusted for adiposity measures.

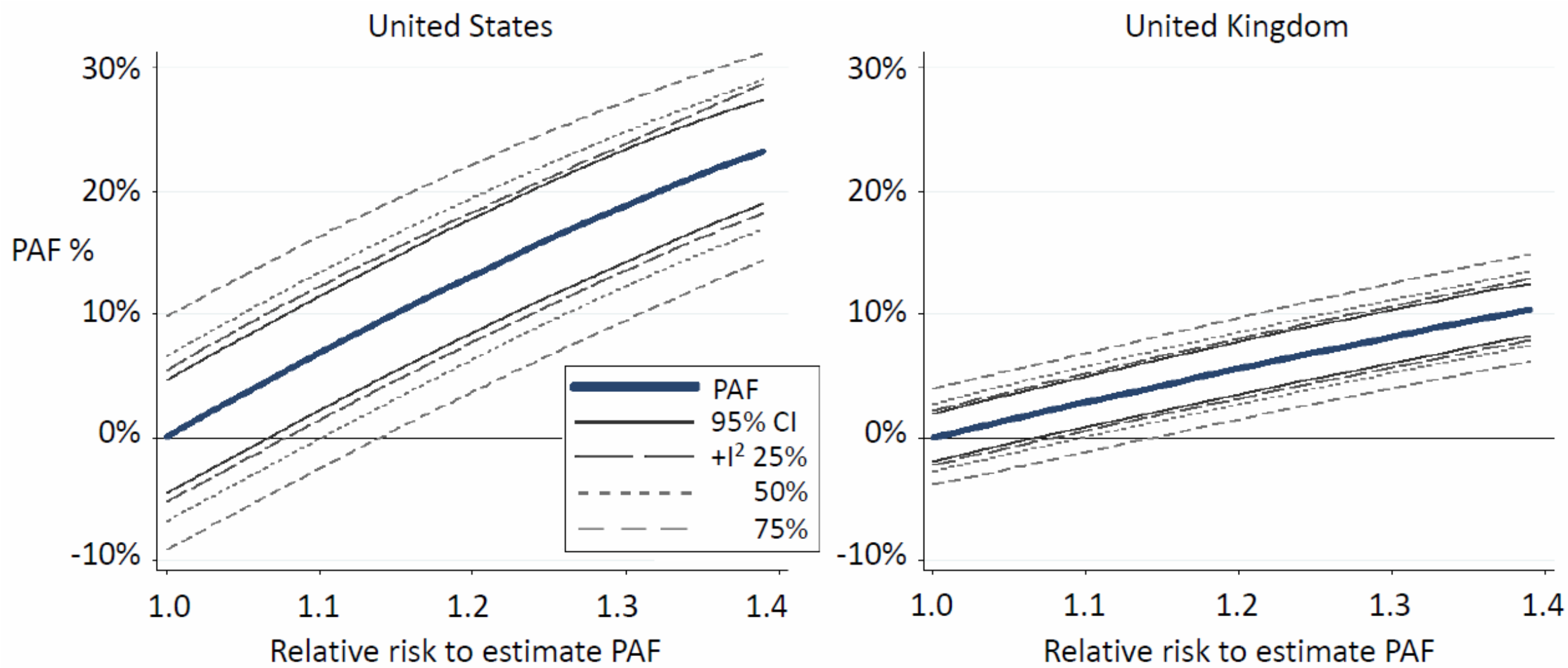

Figure S5. Population attributable fraction (PAF) for different degrees of associations between consumption of sugar sweetened beverages (SSB) and incidence of type 2 diabetes in the United States and the United Kingdom: Sensitivity analyses.

Blue thick line is the best estimates of PAF; thin solid line, 95% confidence interval (CI) of the point estimate with a fixed standard error adjusted for adiposity; and dashed lines further incorporated measures of heterogeneity of potential effect across populations (I^2=25%, 50%, 75%). I^2 was 23.4% after controlling for measured characteristics of populations and identified studies. (age, sex, absolute incidence, body-mass index, location of studies, methods for diabetes ascertainment, measures of validity and reproducibility of dietary assessment).

Supplementary Text

Search Strategy

We undertook electronic searches, using the Internet browser (Firefox 27.0.1). We initially searched existing relevant reviews available at Cochrane Library, Centre for Reviews and Dissemination, Systematic Review Data Repository, PubMed, and OVID. We identified 15 reviews directly related to the topic, and then hand-searched potentially eligible publications for this meta-analysis. To identify additional studies, we systematically searched electronic databases, using specific search terms as described below. No restriction of time or language was applied. This search was performed on May 31, 2013, and repeated on February 10, 2014. We identified all articles included in prior meta-analyses[85–87] and additional studies. Addition of "fizzy", "artificially sweetened beverages" did not change the search results.

OVID and Embase: (("soda" OR "pop" OR "juices" OR "juice" OR "drink" OR "drinks" OR "beverage" OR "beverages") and ("diabetes") and ("prospective" OR "longitudinal" OR "cohort" OR "cohorts" OR "follow-up" OR "case-cohort" OR "nested case-control")) in abstract, title, and sub-headings; 599 hits on May 31, 2013, 52 hits on Feb 10, 2014

PubMed: ("soda"[tiab] OR "pop"[tiab] OR "juices"[tiab] OR "juice"[tiab] OR "drink"[tiab] OR "drinks"[tiab] OR "beverage"[tiab] OR "beverages"[tiab] OR "beverage[MeSH]" OR "beverages" [MeSH]) and ("diabetes"[tiab] or "diabetes" [MeSH]) and ("prospective"[tiab] OR "longitudinal"[tiab] OR "cohort"[tiab] OR "cohorts"[tiab] OR "follow-up"[tiab] OR "nested case-control"[tiab] OR "case-cohort"[tiab] OR "Prospective Studies"[Mesh] OR "Cohort Studies"[Mesh] OR "Longitudinal Studies"[Mesh]); 477 hits on May 31, 2013, 30 hits on Feb 10, 2014

Web of Knowledge: Topic=(juice* OR beverage* OR drink OR drinks OR soda OR pop) AND Topic=(diabetes) AND Topic=(prospective OR longitudinal OR cohort* OR follow-up OR case-cohort OR nested case-control); 1556 hits on May 31, 2013, 94 hits on Feb 10, 2014

Open Grey: (juices OR juice OR drink OR drinks OR beverage OR beverages OR soda OR pop) AND (diabetes) AND (prospective OR longitudinal OR cohort OR cohorts OR follow-up OR case-cohort OR nested case-control); 0 hit on May 31, 2013, 0 hits on Feb 10, 2014

Identification of studies and contact to authors

The articles reviewed in full-text are presented in **Table S1**. From each cohort, we hand-searched multiple publications and examined availability of information on dietary consumption and incident T2D. We contacted authors of the identified articles between October and December in 2013 and requested information needed for this meta-analysis to minimize publication bias. If a publication reported estimates based on either continuous or categorical variables of beverage consumption for both adiposity-adjusted and unadjusted associations, we did not request additional data. However, when we requested any additional information, we requested both continuous and categorical estimates. We sent a reminder two weeks after a contact, in case of no reply.

Quality assessment

We collected information to identify potential bias, in concordance with A Cochrane Risk of Bias Assessment Tool[88] and for Non-Randomized Studies of Interventions (ACROBAT-NRSI)[89]. As instructed by the Bias Assessment Tool[88], a 'high', 'low', or 'unknown' risk of bias in each study was assigned to seven different bias domains and overall risk of bias (**Table S2**). Considerations of overall bias are written here, followed by those for bias in seven domains. Influence of potential sources of bias was examined in sensitivity analyses by testing heterogeneity due to presence or absence of bias, by performing meta-analysis after excluding studies with a certain type of bias (**Table S5**, **Table S6**), or by incorporating quantitative bias in meta-analysis (see below). Influence of a single study can be inferred in influence analysis (**Figure S3**).

- Overall bias: We acknowledged ACRBAT-NRSI's recommendation that an observational study is not likely to have 'low' risk of bias.[89] Then, we assigned 'high' or 'unknown' risk of bias to each study.[88] We considered whether or not multiple sources of bias would impact estimated effects and uncertainty. We did not assign 'high' risk of bias even when studies were likely to have domain-specific bias, if the sources of bias were not likely to impact study estimates or uncertainty substantially and plausibly. Thus, 'unknown' overall bias was assigned to several studies, although they might have bias in some domains[26,31,41,48,49,52–54], because there was no strong plausibility that bias caused substantial impact on effect estimates to be used in this meta-analysis. Studies rated to have 'high' risk of bias had <20% of weights in the main meta-analyses, and exclusion of these studies did not change results (see sensitivity analysis, below).

Here, we describe considerations of seven domains: confounding, selection, measurement errors, misclassification of exposure, missing data, outcome assessment, and selective reporting. Influence of a single source of bias on overall risk of bias is also described. A single source of bias was not necessarily considered influential on overall bias as described above, with regards to biological plausibility and sensitivity analyses.

- Confounding: Residual confounding is likely in any of observational research. Thus, a 'low' risk of bias was not assigned to any studies, as anticipated.[89] A 'high' risk was assigned to the E3N cohort, which was likely to have residual confounding by adiposity in analysis of ASB.[32,43–45] Potential confounders adjusted for in each study are summarized in **Table S4**. With exception of adiposity measures, five studies[14–19,31,36] confirmed little influences of bias due to confounding by each of socio-demographic variables, lifestyle factors, and clinical variables (family history of diabetes, mediaction use, and prevalent disaeses), in a multivariable model specified in each study. However, comparison between crude and adjusted analyses indicates confounding in analysis of each beverage, particularly of ASB (**Table S4; Table 2**).
- Selection: Selection of participants into a study would cause bias, if selection was related to both beverage consumption and incidence of T2D. This possibility was not identified in any of the studies. Selection was partly based on completion of data in any studies and considered in the assessment of the domain of missing data (see below). We considered that 'high' risk of bias of this point would not necessarily influence overall quality of point estimates. On the other hand, we assigned 'high' risk of bias to Atherosclerosis Risk in Communities Study (ARIC) and Singapore Chinese Health Study (SCHS), because of misclassification of beverage types. Because influence of this particular bias on study results was likely to be present, 'high' risk of overall bias was rated for these two studies.
- Measurement errors: Because any dietary assessments involve measurement errors, any studies had risks of this bias. However, inclusion in this meta-analysis incorporated the quantity of the bias partly (see next subsection). Accounting for it, a 'high' risk of bias was assigned to studies that did not verify quality of a diet-assessment method within a study population[23,25,26,49,52–54,59] or studies that assessed diets during a month or less and did not confirm long-term reproducibility of dietary measures.[12,51,52]
- Misclassification of exposure: We focused on exposure assessment during follow-up. 'Low' risk of bias was assigned to studies that assessed dietary exposure repeatedly and incorporated them in analysis.[14–17,26,35] By contrast, 'high' risk was assigned to studies relying on baseline dietary measurements. We regarded that this bias was not likely to influence results substantially, generally causing bias in false-negative findings.
- Missing data: All studies excluded participants with missing information. The number of participants excluded was not large in each study, and the exclusion was considered to be unlikely to cause bias. 'High' risk was assigned to two studies[12,34] because participants were excluded based on missing outcomes, which might cause attrition bias, during the follow-up: deaths in SCHS (15% of adults) and unknown loss to follow-up in the High-risk and Population Strategy for Occupational Health Promotion Study (HIPOP-OHP, 31% of adults). While SCHS excluded prevalent cases of cardiovascular diseases and cancer, HIPOP-OHP recruited adults in a population at high risk of deaths due to cardiometabolic diseases. The latter was considered as having high risk of overall bias due to missing outcome related to the association of interest.
- Outcome assessment: 'Low' risk was assigned to studies that attempted to minimize both false-positive and false-negative cases in a whole cohort by using objective information on incidence of T2D. 'High' risk would have been assigned if a differential misclassification had occurred. This bias was not indicated in any cohorts. Coronary Artery Risk Development in Young Adults Study (CARDIA) was rated as having 'high' risk of this domain: CARDIA examined a prospective association of beverage consumption with a risk of developing hyperglycaemia that included also incident type 2 diabetes; combined with selective reporting (see the next point), the overall bias in this study was rated as high.
- Selective reporting: 'High' risk was assigned to two studies, on the basis of multiple analyses in different sub-groups, that presented inconsistent methods across articles from each cohort for similar research questions.[21,22,28,30]. These two studies, including CARDIA, were considered as having high risk of bias, because of uncertainty if main effect estimates were selected validly. 'High' risk was also assigned to the other four studies that reported estimates of associations selectively on the basis of whether or not findings were significant[23,25,35,36], but each presentation was judged as independent of validity of effect estimates. 'Unknown' risk of bias was assigned to four studies which we requested unpublished estimates of beverage-diabetes associations. We did not find plausible explanation that each reporting led to biased estimates, and therefore we considered that this component is important to highlight, but did not affect overall risk of bias.

Adjustment for within-person variation of beverage consumption

In epidemiologic studies on dietary habits and other exposure related to chronic diseases, random within-person variability is concerning as a source of bias.[90] We applied statistical correction for the potential bias, using measures of the within-person variability, in addition to false-positive ascertainment of self-reported T2D (**Table S3**)[91,92]. Information extracted and assumptions are presented here in compliance with PRISMA.

We extracted correlation coefficients (*r*) between estimates from the two methods compared; ratios of two standard deviations (SDs) from the two dietary methods (s_{obs}/s_{ref}); and sample sizes (*n*). For studies without these measures derived within a study population[13,20,23,25,38], we extracted information from external sources, assuming consistency of within-person variations of dietary assessments in different cohorts. [90,93–98] This assumption was supported by prior research comparing different FFQs in an independent population[67,99] and also by Atherosclerosis Risk in Communities Study (ARIC) that confirmed estimates of sugar intakes by FFQ externally developed were correlated with a biomarker of sugar intakes.[100]

Kuopio Ischaemic Heart Disease Risk Factors Study in Finland evaluated beverage consumption by 4-day diet records implemented only at baseline.[63] A single 4-day diet record is unlikely to capture habitual diets.[66] Thus, we assumed similarity between *r* of single 4-day diet records and *r* of a seasonal variation of diet within a year; and took the measure from another study assessing diets among men in North Sweden[52] selected by demographic similarity.[63,67] We also assumed no error in a between-individual SD in the cohort (s_{obs}/s_{ref}, =1.0).

European Investigation into Cancer and Nutrition Study (EPIC)-InterAct carried out analyses pooling cohorts across Europe.[31,33] We extracted within-person variations of participating cohorts[42,52,69–78] (available on request) and pooled estimates by weights contributing to EPIC-InterAct's estimates[31], averaging *r* and s_{obs}/s_{ref} after *Z*- and log-transformation.[67,68]

When there was no information on measures of validity specifically for each type of beverages, we used information on foods or nutrients that were likely to have similar measures of the variations of consumption, as performed on another topic.[101] For example, if only non-alcohol beverages were assessed, we used them. If nutrients, not foods, were assessed, we extracted information on sucrose, disaccharides or total carbohydrates as surrogates for SSB and ASB; these sugars are not in ASB, but we assumed similarity in within-person dietary variations between ASB consumption and sugar intake.

We adopted a model used in prior meta-analyses[90,93–98] to adjust diet-disease association for a within-person dietary variation: $f\,(risk)=\alpha+\beta_{true}\cdot x_{true}$ and $\beta_{true}=\beta_{obs}/\gamma$, where x_{true} is true dietary factor and β_{true} is unobserved, unbiased log(RR) without a within-person variation (σ^2_{within}). β is attenuated to be β_{obs} by degree of γ, a attenuation factor, representing a variance ratio: $\sigma^2_{within}/(\sigma^2_{within}+\sigma^2_{between})$. In each cohort, γ was calculated by $\gamma=s_{ref}\,/\,s_{obs}\cdot r$, given a linear regression of $x_{true}=\alpha+\beta\cdot x_{obs}$. Dietary habits were measured repeatedly in six cohorts to minimize regression dilution or a degree of attenuation (**Table S2**; **Table S3**).[14,16–19,21,22,27,35] To account for this, γ was recalibrated for the number of repeated measures and measures of reproducibility[90]. Measures of reproducibility were obtained from existing literature along with those of validity (data not shown).

Adjustment for precision of incident type 2 diabetes
Some studies used self-reported T2D only[15–19,23,28,35,102] (**Table S3**), raising possibility of false-positive diagnosis expressed as positive predictive value (PPV). Thus, correction for PPV<1.0 was applied.[91,92] We assumed PPV=1 for studies using objective measures of T2D diagnosis. In CARDIA. two studies on beverages[21,26] ascertained cases with hyperglycaemia, not T2D. Thus, calibration in CARDIA was applied throughout in the meta-analysis, assigning PPV as a proportion of T2D cases among those with incident hyperglycemia[57,62]

Assessment of heterogeneity
Meta-regression was used to assess potential sources of heterogeneity (**Table S5**). Estimates used were those adjusted for adiposity, within-person dietary variation, and precision of T2D; results were similar in post hoc meta-regression using estimates without adjustment for within-person dietary variations (data not shown). Variables assessed by meta-regression were pre-specified, including study-specific factors: geographical location (the United States or Europe, or Asia, categorized *post hoc*), average age (years), sex (% men), average BMI (kg/m^2), follow-up duration (years), absolute risk of T2D (cases / person-years), methods of dietary assessments (FFQ, diet history), and methods of T2D diagnosis (self-reported, others). Publication status (peer-reviewed or not), selective reporting (yes or no), and mutual adjustment for three beverage types were assessed *post hoc*, identified to be potentially important after data extraction. In stratified analysis for a continuous variable, a median across identified cohorts was used.

Independent sources of heterogeneity were selected by meta-regression with forward-variable selection. If variables in meta-regression showed *P*<0.20, the variable with the lowest *P*-value was retained in the model. Then, adjusting for the variable retained, mete-regression was repeated for remaining variables. If any of additional variables did not produce *P*<0.20, the model was considered best fitted. A variable with *P*<0.10 was considered as a significant source of heterogeneity and meta-analysis stratified by the factor was performed.[103]

Sensitivity analysis
We performed sensitivity analysis to confirm robustness of our findings against decision of modelling, assumptions and different use of available information (**Table S6)**. Sensitivity analysis included influence analysis[46] by excluding a single study and repeating random-effects meta-analysis (**Figure S3**). We also took an iterative stochastic sensitivity analysis, accounting for additional uncertainty of adjustment for within-person variations and precision of T2D diagnosis.[46,79,82] Therefore, to confirm stability of our main analysis, we repeated the main meta-analysis (10,000 times) after ln(RR), γ and PPV were randomly drawn from each distribution determined by each central estimate and standard errors (SE). SE of ln(RR) was obtained by dose-response estimation; SE of γ, derived from information available in published records assessing within-person variations; SE of PPV, derived from 95% confidence interval (CI) calculated by Wilson score interval[104] or, when PPV=1, the rule of three.[105] In each iteration, trim-and-fill analysis was applied, assuming these estimates were the least subject to bias.[106] Medians of ln(RR) and 95% confidence limits of ln(RR) were used as the estimate accounting for uncertainty of our approach.[82]

Sensitivity analysis for residual confounding
We included a simulation-based analysis to examine influence of residual confounding. We recognized the limitation of the abovementioned correction for within-person variations of the main exposure, but not of confounders.[6] Residual confounding by within-person variation of adiposity measures would be expected and crucial source of bias.[31,107] Thus, adjustment for the bias could have been done, using measures of the within-person variation of adiposity and associations of adiposity with beverage consumption and incident T2D. Because the information was not available in any studies, we undertook simulation extrapolation (SIMEX), an imperfect, but useful, technique when structure of measurement errors is likely to be complex or unknown.[84]

In SIMEX, we used estimates after adjustment for potential publication bias by trim-and-fill method, assuming these estimates were the least subject to bias. Inference became similar without trim-and-fill method (data not shown). In SIMEX, first, we assumed that adiposity was measured with within-person variation (r_a): when r_a=1.0, observed ln(RR) adjusted for adiposity would be unbiased; when r_a=0, adiposity measures would be a random variable and ln(RR) would be ln(RR) unadjusted for adiposity. We assumed r_a =0.9, 0.8, and 0.7 based on a study assessing validity of adiposity measures.[83]

We assumed that ln(RR) adjusted for measured adiposity could be expressed as $ln(RR) = \alpha + \beta \cdot tanh^{-1}(r_a)$ (Model A). This model supports that, r_a=0 would make ln(RR) equivalent to ln(RR) unadjusted for adiposity. Then, α and β could be readily solved algebraically by r_a, observed ln(RR) unadjusted for adiposity measures, and observed ln(RR) adjusted for adiposity measures.

Separately, a non-linear SIMEX formula was modelled[84]: $ln(RR) = a + 1/(b + c\theta)$ (Model B), where *a*, *b*, and *c* were constants; and θ was a degree of within-person variations of measured adiposity, following $r_a = \frac{\sigma_{between}}{\sigma_{between}+\sigma_{within}(1+\theta)}$. This denotes that, when θ=0, ln(RR) was ln(RR) adjusted for adiposity, given r_a assumed; and when θ=∞, ln(RR) would be unadjusted for adiposity measures.

We used the Model A and B developed with r_a={0.9, 0.8, 0.7} and related ln(RR) to θ. We then obtained *a*, *b* and *c* based on θ={0, 0.5, 1.0, 2.0}. Finally, using $ln(RR) = a + 1/(b + c\theta)$, we extrapolated ln(RR) of $\theta = -1$, producing $\sigma_{within} = 0$ and ln(RR) corrected for mis-measurements of adiposity.[84]

Estimation of type 2 diabetes events over 10 years from 2010 attributable to consumption of sugar-sweetened beverages in the United States and in the United Kingdom.
We estimated population attributable fraction (PAF) for T2D due to SSB consumption. We evaluated adults aged 20 years or older and free of T2D who participated in each of the national dietary surveys: US National Health and Nutrition Examination Survey (US NHANES), 2009-2010[108]; and the UK National Dietary Nutrition Survey (UK NDNS), 2008/2009-2011/2012[109]. The contemporary national surveys strengthen the implication from the present meta-analysis, beyond estimation solely relying on cohorts limited in generalizability of a source population.[110] Moreover, the use of individuals' data can avoid some assumption often adopted in analysis of PAF and other estimates of social impact due to dietary exposure, for example implausible assumption of normal distribution of dietary consumption.[110–112]

Overall, in each survey, we (1) estimated habitual consumption of SSB among adults; (2) predicted 10-year risk ('assumed control risk', *ACR*[113]) of developing T2D of each adult; (3) estimated separate ideal 10-year risk (R_i) for each adult if SSB consumption was reduced to zero; and (4) estimated (*ACR*–R_i) for each adult and Σ(*ACR*-

R_i) × population size as a number of cases attributable to SSB consumption in a population. PAF was derived as Σ(ACR-R_i)/ Σ(ACR).

As a simple example, if one adult consumed 1 serving/day of SSB and had ACR of 0.10 and if SSB consumption became zero, his or her risk (R_i) would be 0.10/1.13=0.088, where 1.13 is RR adjusted for adiposity. This calculation was applied to all adults, and pooling them as Σ(ACR) and Σ(R_i), the population-based estimates were obtained.[110] This estimation has advantage that there is no need of assumption in exposure distribution.

We used two RR separately: RR unadjusted for adiposity and RR adjusted for adiposity (1.18 and 1.13, respectively). We did not use RR unadjusted for within-person dietary variation, because the 10-year risk prediction was based on T2D risk factors unadjusted for within-person variations; and because reduction of SSB consumption in a population is likely to occur, involving a random within-person fluctuation. In addition to uncertainty in this probability-weighting analysis, the uncertainty of RR was incorporated by one thousand iteration varying RR as normally distributed with variance of ln(RR). Potential lack of generalisability of RR and heterogeneity of RR were secondarily examined in sensitivity analysis for PAF.

The next subsections describe each estimation of PAF in the US and the UK; validation analysis implemented by using the US observations; and sensitivity analysis varying RR and incorporating I^2 to calculate PAF.

Population attributable fraction for T2D due to SSB in the United States

In the US NHANES, we evaluated 4,729 non-diabetic adults who represented 189,075,538 adults in the US 2009-2010 according to sampling probability, after excluding 5,928 individuals: 4,319 children and adolescents (age<20 years) and 1,033 adults with prevalent diabetes (13.7% in weighted analysis) defined by reported diagnosis or anti-diabetic drug use or by fasting glucose ≥7.0 mmol/L or glycated haemoglobin ≥6.5%.[114]

Habitual consumption of SSB was estimated by using two 24-hour recall and a dietary screener questionnaire simultaneously analysed through a method to minimize within-individual dietary variation.[115] Using a set of risk factors (z) for T2D, 10-year risk of T2D was estimated by a risk-prediction algorithm developed in ARIC and validated in Multiethnic Study on Atherosclerosis (MESA), a community-based cohort in US.[116,117] The formula was a logistic function, $ACR=1/(1+exp(-X|z))$. The original prediction was for a 9-year risk of T2D, and thus converted to a 10-year risk as $Pr=1-(1-Pr)^{10 \div 9}$. The model was developed among adults younger than 65 years, adopting a rare-disease assumption. Mortality, reducing T2D cases identified over 10 years, was accounted by age-sex-specific mortality due to non-diabetes cause $(1\text{-annual mortality})^{10 \text{ years}}$, based on US vital statistics.[118]

Population attributable fraction for T2D due to SSB in the United Kingdom

We used the UK NDNS data collected in 2008/2009-2011/2012.[109] Sampling weights were applied, which appeared, unlike US NHANES, not to estimate an absolute number of adults with a certain condition in UK. Thus, to estimate absolute numbers of T2D cases attributable to SSB, we used age-sex-specific population sizes in UK in 2010 as the source population (47,704,520 adults in total, aged 20 or older).[119] Of the UK NDNS, we evaluated 1,932 non-diabetic adults, after excluding 2,096 children and adolescents (age<20 years) and 128 adults with prevalent diabetes (6.2% in weighted analysis, 2.9 million in UK) defined by diagnosis, anti-diabetic drug use, or compliance to an anti-diabetic diet assessed through an interview; or by fasting glucose ≥7.0 mmol/L or glycated haemoglobin ≥6.5%.

Habitual consumption of SSB was estimated by 4-day food records with within-individual dietary variation minimized.[115] Ten-year risk of T2D was estimated by a risk-prediction algorithm, QDScore®-2013[120], developed in the prospective analysis of nation-wide electronic records collected in UK general practice; validated externally[121,122]; and made publically available for research purpose.[120] The formula allowed estimation of ACR over 10 years from basic demographic variables, deprivation index, smoking status, use of an oral corticosteroid, use of an anti-hypertensive drug, prevalent cardiovascular diseases, family history of diabetes.[120–122] Family history of diabetes and Townsend deprivation index were not available in NDNS. These were imputed, respectively, by the population average as found in the nation-wide electronic record[121] and by household income, as recommended previously[121].

Validation of 10-year risk prediction

The present estimates showed that the US population had approximately two-fold greater SSB consumption, T2D prevalence, and T2D risk compared to the UK population (**Table S7**). These ecological differences were comparable to those observed in prior international studies[123,124]. In addition, 10-year risk in US and UK were comparable to previous population-based estimates.[116,117,121,122] These partly supported validity of our estimates.

We assessed validity of 10-year risk prediction in US NHANES, using the 1999-2000 and 2009-2010 cycles. We first estimated prevalent T2D predicted by NHANES 1999-2000. Adults with T2D in 1999-2000 were assumed to have T2D in 2009-2010, with Pr= (1-annual mortality)^{10}. For non-diabetic adults, 10-year risk prediction was applied as described above. Then, the two numbers of T2D cases from T2D cases and non-cases in 1999-2000 were summed as the predicted number of T2D cases in 2009-2010. The sum was compared to observed number of cases in 2009-2010. The two estimates were not statistically different (*P*=0.48). The number of T2D cases in 2009-2010 predicted by NHANES 1999-2000 was 32.1 million [95% CI=27.1-37.1]); and that observed in NHANES 2009-2010 was (30.0 million [95% CI=26.7-33.3]). Thus, we considered validity of 10-year risk prediction to be sufficient in this work.

Sensitivity analysis varying relative risk and incorporating its heterogeneity to estimate PAF
PAF is generally estimated on the basis of an association of exposure with an outcome. The measure of association often has heterogeneity across populations across measured or unmeasured factors; and uncertainty in its generalisability to a target population.[125] To present how PAF may vary and how precise PAF could be across different values of RR, we performed sensitivity analysis by varying RR and incorporating a measure of heterogeneity, I^2 (25%, 50%, and 75%). The standard error (SE) for RR was derived from estimates adjusted for adiposity measure (RR=1.13, 95% CI=1.06-1.21). I^2 was converted to a measure of between-population variance, τ^2, by using $I^2 = \tau^2/(\tau^2 + SE^2)$. We specified RR to be 1.00 to 1.42 incremented by 0.03 (15 values of RRs). Each of RRs simulated 1,000 times to vary by the degree of the pooled variance, $\sqrt{\tau^2 + SE^2}$. For each RR (15 × 1,000), PAF was calculated by using each of the US and the UK datasets. 95% CI of PAF across RRs was based on 2.5th and 97.5th percentiles of 1,000 repeats.[125] The results are displayed in **Figure S5**. As expected, the greater I^2, the wider 95% CI.

References of Supplementary Materials
1. Wannamethee G, Shaper AG. Men who do not drink - a report from the British Regional Heart Study. *Int J Epidemiol*. 1988;17(2):307–316.
2. Perry IJ, Wannamethee SG, Walker MK, Thomson a G, Whincup PH, Shaper a G. Prospective study of risk factors for development of non-insulin dependent diabetes in middle aged British men. *BMJ*. 1995;310(6979):560–4.
3. Burke V, Zhao Y, Lee AH, et al. Predictors of type 2 diabetes and diabetes-related hospitalisation in an Australian Aboriginal cohort. *Diab Res Clin Pr*. 2007;78(3):360–368.
4. Gurwitz JH, Field TS, Glynn RJ, et al. Risk-factors for non-insulin-dependent diabetes mellitus requuiring treatment in the elderly. *J Am Geriatr Soc*. 1994;42(12):1235–1240.
5. Abbasi A, Corpeleijn E, van der Schouw YT, et al. Maternal and paternal transmission of type 2 diabetes: influence of diet, lifestyle and adiposity. *J Intern Med*. 2011;270(4):388–396.
6. Kiyohara Y, Shinohara A, Kato I, et al. Dietary factors and development of impaired glucose tolerance and diabetes in a general Japanese population: The Hisayama study. *J Epidemiol*. 2003;13(5):251–258.
7. Barrio-Lopez MT, Martinez-Gonzalez MA, Fernandez-Montero A, Beunza JJ, Zazpe I, Bes-Rastrollo M. Prospective study of changes in sugar-sweetened beverage consumption and the incidence of the metabolic syndrome and its components: the SUN cohort. *Br J Nutr*. 2013:1–10.
8. Janket SJ, Manson JE, Sesso H, Buring JE, Liu SM. A prospective study of sugar intake and risk of type 2 diabetes in women. *Diabetes Care*. 2003;26(4):1008–1015.
9. Djoussé L, Driver JA, Gaziano JM, Buring JE, Lee IM, Djousse L. Association between modifiable lifestyle factors and residual lifetime risk of diabetes. *Nutr Metab Cardiovasc Dis*. 2013;23(1):17–22.
10. Reis JP, Loria CM, Sorlie PD, Park Y, Hollenbeck A, Schatzkin A. Lifestyle Factors and Risk for New-Onset Diabetes A Population-Based Cohort Study. *Ann Intern Med*. 2011;155(5):292–299.
11. Roussel R, Fezeu LL, Bouby N, et al. Low Water Intake and Risk for New-Onset Hyperglycemia. *Diabetes Care*. 2011;34(12):2551–2554.
12. Hayashino Y, Fukuhara S, Okamura T, et al. A prospective study of passive smoking and risk of diabetes in a cohort of workers: the High-Risk and Population Strategy for Occupational Health Promotion (HIPOP-OHP) study. *Diabetes Care*. 2008;31(4):732–734.
13. Montonen J, Jarvinen R, Knekt P, Heliovaara M, Reunanen A. Consumption of sweetened beverages and intakes of fructose and glucose predict type 2 diabetes occurrence. *J Nutr*. 2007;137(6):1447–1454.
14. Muraki I, Imamura F, Manson JE, et al. Fruit consumption and risk of type 2 diabetes: results from three prospective longitudinal cohort studies. *BMJ*. 2013;347:f5001.
15. Bazzano LA, Li TY, Joshipura KJ, Hu FB. Intake of fruit, vegetables, and fruit juices and risk of diabetes in women. *Diabetes Care*. 2008;31(7):1311–1317.
16. Bhupathiraju SN, Pan A, Malik VS, et al. Caffeinated and caffeine-free beverages and risk of type 2 diabetes. *Am J Clin Nutr*. 2013;97(1):155–166.
17. De Koning L, Malik VS, Rimm EB, Willett WC, Hu FB. Sugar-sweetened and artificially sweetened beverage consumption and risk of type 2 diabetes in men. *Am J Clin Nutr*. 2011;93(6):1321–1327.
18. Schulze MB, Manson JE, Ludwig DS, et al. Sugar-sweetened beverages, weight gain, and incidence of type 2 diabetes in young and middle-aged women. *JAMA*. 2004;292(8):927–34.
19. Pan A, Malik VS, Schulze MB, Manson JE, Willett WC, Hu FB. Plain-water intake and risk of type 2 diabetes in young and middle-aged women. *Am J Clin Nutr*. 2012;95(6):1454–1460.
20. Mursu J, Virtanen JK, Tuomainen T-P, Nurmi T, Voutilainen S. Intake of fruit, berries, and vegetables and risk of type 2 diabetes in Finnish men: the Kuopio Ischaemic Heart Disease Risk Factor Study. *Am J Clin Nutr*. 2014;99(2):328–33.

21. Duffey KJ, Gordon-Larsen P, Steffen LM, et al. Drinking caloric beverages increases the risk of adverse cardiometabolic outcomes in the Coronary Artery Risk Development in Young Adults (CARDIA) Study. *Am J Clin Nutr*. 2010;92(4):954–959.
22. Duffey KJ, Steffen LM, Van Horn L, Jacobs DR, Popkin BM. Dietary patterns matter: diet beverages and cardiometabolic risks in the longitudinal Coronary Artery Risk Development in Young Adults (CARDIA) Study. *Am J Clin Nutr*. 2012;95(4):909–15.
23. Pereira MA, Parker ED, Folsom AR. Intake of sugar sweetened beverages, fruit juice, and incidence of type 2 diabetes: A prospective study of postmenopausal women. *Diabetes*. 2005;54:A258–A258.
24. Meyer KA, Kushi LH, Jacobs DR, Slavin J, Sellers TA, Folsom AR. Carbohydrates, dietary fiber, and incident type 2 diabetes in older women. *Am J Clin Nutr*. 2000;71(4):921–30.
25. Paynter NP, Yeh H-CC, Voutilainen S, et al. Coffee and sweetened beverage consumption and the risk of type 2 diabetes mellitus: the atherosclerosis risk in communities study. *Am J Epidemiol*. 2006;164(11):1075–84.
26. Dhingra R, Sullivan L, Jacques PF, et al. Soft drink consumption and risk of developing cardiometabolic risk factors and the metabolic syndrome in middle-aged adults in the community. *Circulation*. 2007;116(5):480–488.
27. Jacques PF, Cassidy A, Rogers G, Peterson JJ, Meigs JB, Dwyer JT. Higher dietary flavonol intake is associated with lower incidence of type 2 diabetes. *J Nutr*. 2013;143(9):1474–80.
28. Eshak ES, Iso H, Mizoue T, Inoue M, Noda M, Tsugane S. Soft drink, 100% fruit juice, and vegetable juice intakes and risk of diabetes mellitus. *Clin Nutr*. 2013;32(2):300–308.
29. Hu FB, Stampfer MJ, Rimm E, et al. Dietary fat anc coronary heart disease: a comparison of approaches for adjusting for total energy intake and modeling repeated dietary measurements. *Am J Epidemiol*. 1999;149(6):531–540.
30. Eshak ES, Iso H, Kokubo Y, et al. Soft drink intake in relation to incident ischemic heart disease, stroke, and stroke subtypes in Japanese men and women: the Japan Public Health Centre-based study cohort I. *Am J Clin Nutr*. 2012;96(6):1390–7.
31. The InterAct Consortium. Consumption of sweet beverages and type 2 diabetes incidence in European adults: results from EPIC-InterAct. *Diabetologia*. 2013;56(7):1520–1530.
32. Fagherazzi G, Vilier A, Saes Sartorelli D, Lajous M, Balkau B, Clavel-Chapelon F. Consumption of artificially and sugar-sweetened beverages and incident type 2 diabetes in the Etude Epidemiologique aupres des femmes de la Mutuelle Generale de l'Education Nationale-European Prospective Investigation into Cancer and Nutrition cohort. *Am J Clin Nutr*. 2013;97(3):517–23.
33. The InterAct Consortium. Design and cohort description of the InterAct Project: an examination of the interaction of genetic and lifestyle factors on the incidence of type 2 diabetes in the EPIC Study. *Diabetologia*. 2011;54(9):2272–2282.
34. Odegaard AO, Koh W-PP, Arakawa K, Yu MC, Pereira M a. Soft drink and juice consumption and risk of physician-diagnosed incident type 2 diabetes: the Singapore Chinese Health Study. *Am J Epidemiol*. 2010;171(6):701–708.
35. Palmer JR, Boggs DA, Krishnan S, Hu FB, Singer M, Rosenberg L. Sugar-sweetened beverages and incidence of type 2 diabetes mellitus in African American women. *Arch Intern Med*. 2008;168(14):1487–92.
36. Nettleton JA, Lutsey PL, Wang Y, et al. Diet soda intake and risk of incident metabolic syndrome and type 2 diabetes in the Multi-Ethnic Study of Atherosclerosis (MESA). *Diabetes Care*. 2009;32(4):688–694.
37. Malik VS, Hu FB. Sweeteners and Risk of Obesity and Type 2 Diabetes: The Role of Sugar-Sweetened Beverages. *Curr Diabetes Rep*. 2012;12(2):195–203.
38. Sakurai M, Nakamura K, Miura K, et al. Sugar-sweetened beverage and diet soda consumption and the 7-year risk for type 2 diabetes mellitus in middle-aged Japanese men. *Eur J Nutr*. 2014;53(1):251–8.
39. Hu FB, Rimm E, Smith-warner SA, et al. Reproducibility and validity of dietary patterns assessed with a food-frequency questionnaire. *Am J Clin Nutr*. 1999;69(2):243–249.
40. Feskanich D, Rimm EB, Giovannucci EL, et al. Reproducibility and validity of food intake measurements from a semiquantitative food frequency questionnaire. *J Am Diet Assoc*. 1993;93(7):790–796.
41. Kumanyika S. Relative Validity of Food Frequency Questionnaire Nutrient Estimates in the Black Women's Health Study. *Ann Epidemiol*. 2003;13(2):111–118.
42. Van Liere MJ, Lucas F, Clavel F, Slimani N, Villeminot S. Relative validity and reproducibility of a French dietary history questionnaire. *Int J Epidemiol*. 1997;26(suppl_1):S128–136.
43. Fantino M. Consumption of artificially and sugar-sweetened beverages and incident type 2 diabetes: methodologic concern about a recent epidemiological study. *Am J Clin Nutr*. 2013;98(1):251–2.
44. Stevens HC. Diabetes and diet beverage study has serious limitations. *Am J Clin Nutr*. 2013;98(1):248–9.
45. La Vecchia C. Artificially and sugar-sweetened beverages and incident type 2 diabetes. *Am J Clin Nutr*. 2013;98(1):249–50.
46. Greenland S. Quantitative methods in the review of epidemiologic literature. *Epidemiol Rev*. 1987;9(1):1–30.
47. Hankin JH, Stram DO, Arakawa K, et al. Singapore Chinese Health Study: development, validation, and calibration of the quantitative food frequency questionnaire. *Nutr Cancer*. 2001;39(2):187–95.
48. Tsubono Y, Kobayashi M, Sasaki S, Tsugane S. Validity and reproducibility of a self-administered food frequency questionnaire used in the baseline survey of the JPHC Study Cohort I. *J Epidemiol*. 2003;13(1 Suppl):S125–33.
49. Kobayashi S, Murakami K, Sasaki S, et al. Comparison of relative validity of food group intakes estimated by comprehensive and brief-type self-administered diet history questionnaires against 16 d dietary records in Japanese adults. *Public Heal Nutr*. 2011;14(7):1200–11.
50. Chiba N, Okuda N, Okayama A, Kadowaki T, Ueshima H. Development of a Food Frequency and Quantity Method for Assessing Dietary Habits of Japanese Individuals -Comparison with Results from 24hr Recall Dietary Survey. *J Atheroscler Thromb*. 2008;15(6):324–333.
51. Liu K, Slattery M, Jacobs D, et al. A study of the reliability and comparative validity of the cardia dietary history. *Ethn Dis*. 1994;4(1):15–27.
52. Johansson I, Hallmans G, Wikman A, Biessy C, Riboli E, Kaaks R. Validation and calibration of food-frequency questionnaire measurements in the Northern Sweden Health and Disease cohort. *Public Health Nutr*. 2002;5(3):487–96.

53. Van Leeuwen FE, de Vet HC, Hayes RB, van Staveren WA, West CE, Hautvast JG. An assessment of the relative validity of retrospective interviewing for measuring dietary intake. *Am J Epidemiol*. 1983;118(5):752–8.
54. Jain M, Howe GR, Johnson KC, Miller AB. Evaluation of a diet history questionnaire for epidemiologic studies. *Am J Epidemiol*. 1980;111(2):212–9.
55. Salvini S, Hunter DJ, Sampson L, et al. Food-based validation of a dietary questionnaire: the effects of week-to-week variation in food consumption. *Int J Epidemiol*. 1989;18(4):858–67.
56. Manson JE, Stampfer MJ, Colditz GA, et al. Physical activity and incidence of non-insulin-dependent diabetes mellitus in women. *Lancet*. 1991;338(8770):774–778.
57. Wilson PWF, D'Agostino RB, Fox CS, Sullivan LM, Meigs JB. Type 2 diabetes risk in persons with dysglycemia: the Framingham Offspring Study. *Diabetes Res Clin Pr*. 2011;92(1):124–7.
58. Hu FB, Leitzmann MF, Stampfer MJ, Colditz GA, Willett WC, Rimm EB. Physical activity and television watching in relation to risk for type 2 diabetes mellitus in men. *Arch Int Med*. 2001;161(12):1542–8.
59. Mayer-Davis EJ, Vitolins MZ, Carmichael SL, et al. Validity and Reproducibility of a Food Frequency Interview in a Multi-Cultural Epidemiologic Study. *Ann Epidemiol*. 1999;9(5):314–324.
60. Odegaard AO, Pereira MA, Koh W-P, Arakawa K, Lee H-P, Yu MC. Coffee, tea, and incident type 2 diabetes: the Singapore Chinese Health Study. *Am J Clin Nutr*. 2008;88(4):979–985.
61. Waki K, Noda M, Sasaki S, et al. Alcohol consumption and other risk factors for self-reported diabetes among middle-aged Japanese: a population-based prospective study in the JPHC study cohort I. *Diabet Med*. 2005;22(3):323–31.
62. He K, Xun P, Liu K, Morris S, Reis J, Guallar E. Mercury exposure in young adulthood and incidence of diabetes later in life: the CARDIA Trace Element Study. *Diabetes Care*. 2013;36(6):1584–9.
63. Mursu J, Tuomainen TP, Virtanen JK, Nurmi T, Voutilainen S. Intake of fruits, berries and vegetables and the risk of type 2 diabetes in finnish men: the Kuopio Ischaemic Heart Disease Risk Factor Study. *Circulation*. 2013;127 (12 Me:2013.
64. Munger RG, Folsom AR, Kushi LH, Kaye SA, Sellers TA. Dietary assessment of older Iowa women with a food frequency questionnaire: nutrient intake, reproducibility, and comparison with 24-hour dietary recall interviews. *Am J Epidemiol*. 1992;136:192–200.
65. Willett WC, Howe GR, Kushi LH. Adjustment for total energy intake in epidemiologic studies. *Am J Clin Nutr*. 1997;65(4 Suppl):1220S–1228S; discussion 1229S–1231S.
66. Willett WC, Lenart E. Reproducibility and Validity of Food Frequency Questionnaires. In: Willett WC, ed. *Nutritional Epidemiology*. 3rd ed. New York, USA: Oxford University Press; 2012.
67. Molag ML, de Vries JHM, Ocké MC, et al. Design characteristics of food frequency questionnaires in relation to their validity. *Am J Epidemiol*. 2007;166(12):1468–78.
68. Rosner B. Regression and correlation methods. In: *Fundamentals of Biostatistics*. 7th ed. Pacific Grove, CA: Duxbury Press; 2010:427–515.
69. Pisani P, Faggiano F, Krogh V, Palli D, Vineis P, Berrino F. Relative validity and reproducibility of a food frequency dietary questionnaire for use in the Italian EPIC centres. *Int J Epidemiol*. 1997;26(suppl_1):S152–160.
70. Bohlscheid-thomas S, Hoting INA, Boeing H, Wahrendorf J. Reproducibility and relative validity of food group intake in a food frequency questionnaire developed for the German part of the EPIC project. European Prospective Investigation into Cancer and Nutrition. *Int J Epidemiol*. 1997;26(suppl 1):S59.
71. Bohlscheid-Thomas S. Reproducibility and relative validity of energy and macronutrient intake of a food frequency questionnaire developed for the German part of the EPIC project. European Prospective Investigation into Cancer and Nutrition. *Int J Epidemiol*. 1997;26(90001):71S–81.
72. Boeing H, Bohlscheid-thomas S, Voss S, Schneeweiss S, Wahrendorf J. The relative validity of vitamin intakes derived from a food frequency questionnaire compared to 24-hour recalls and biological measurements: results from the EPIC pilot study in Germany. European Prospective Investigation into Cancer and Nutrition. *Int J Epidemiol*. 1997;26(suppl_1):S82–90.
73. Ocké MC, Bueno-de-Mesquita HB, Goddijn HE, et al. The Dutch EPIC food frequency questionnaire. I. Description of the questionnaire, and relative validity and reproducibility for food groups. *Int J Epidemiol*. 1997;26(suppl 1):S37.
74. Ocke MC, Bueno-de-Mesquita HB, Pols MA, et al. The Dutch EPIC food frequency questionnaire. II. Relative validity and reproducibility for nutrients. *Int J Epidemiol*. 1997;26(suppl 1):S37.
75. EPIC Group of Spain. Relative validity and reproducibility of a diet history questionnaire in Spain. I. Foods. EPIC Group of Spain. European Prospective Investigation into Cancer and Nutrition. *Int J Epidemiol*. 1997;26(suppl_1):S91–99.
76. EPIC Group of Spain. Relative validity and reproducibility of a diet history questionnaire in Spain. II. Nutrients. EPIC Group of Spain. European Prospective Investigation into Cancer and Nutrition. *Int J Epidemiol*. 1997;26(suppl_1):S100–109.
77. Bingham SA, Gill C, Welch A, et al. Comparison of dietary assessment methods in nutritional epidemiology: weighed records v. 24 h recalls, food-frequency questionnaires and estimated-diet records. *Br J Nutr*. 1994;72(4):619–43.
78. Tjønneland a, OVERVAD K, Haraldsdóttir J, et al. Validation of a Semiquantitative Food Frequency Questionnaire Developed in Denmark. *Int J Epidemiol*. 1991;20(4):906–912.
79. Greenland S, Longnecker MP. Methods for trend estimation from summarized dose-response data, with applications to meta-analysis. *Am J Epidemiol*. 1992;135(11):1301–1309.
80. Palca J. Getting to the Heart of the Cholesterol Debate. *Science (80-)*. 1990;247(4947):1170–1171.
81. MacMahon S, Peto R, Cutler J, et al. Blood pressure, stroke, and coronary heart disease. Part 1, Prolonged differences in blood pressure: prospective observational studies corrected for the regression dilution bias. *Lancet*. 1990;335(8692):765–774.
82. Lash TL, Fink AK. Semi-automated sensitivity analysis to assess systematic errors in observational data. *Epidemiol*. 2003;14(4):451–8.
83. Sun Q, van Dam RM, Spiegelman D, Heymsfield SB, Willett WC, Hu FB. Comparison of dual-energy x-ray absorptiometric and anthropometric measures of adiposity in relation to adiposity-related biologic factors. *Am J Epidemiol*. 2010;172(12):1442–54.

84. Cook JR, Stefanski LA. Simulation-extrapolation estimation in parametric measurement error models. *J Am Stat Assoc*. 1994;89(428):1314–1328.
85. Malik VS, Popkin BM, Bray GA, et al. Sugar-sweetened beverages and risk of metabolic syndrome and type 2 diabetes: a meta-analysis. *Diabetes Care*. 2010;33(11):2477–83.
86. Wang B, Liu K, Mi M, Wang J. Effect of fruit juice on glucose control and insulin sensitivity in adults: a meta-analysis of 12 randomized controlled trials. *PLoS One*. 2014;9(4):e95323.
87. Greenwood DC, Threapleton DE, Evans CEL, et al. Association between sugar-sweetened and artificially sweetened soft drinks and type 2 diabetes: systematic review and dose-response meta-analysis of prospective studies. *Br J Nutr*. 2014;112(5):725–34.
88. Higgins JP, Altman DG, Gotzsche PC, et al. The Cochrane Collaboration's tool for assessing risk of bias in randomised trials. *BMJ*. 2011;343:d5928.
89. Sterne JA, Higgins JP, Reeves BC, on behalf of the development group for ACROBAT-NRSI A. A Cochrane Risk of Bias Assessment Tool: for Non-Randomized Studies of Interventions, Version 1.0.0 September 2014. 2014. Available at: http://www.riskofbias.info.
90. Walter WC. Correction for the Effects of Measurement Error. In: Willett WC, ed. *Nutritional Epidemiology*. 3rd ed. New York, NY: Oxford University Press; 2012.
91. Brenner H, Gefeller O. Use of the positive predictive value to correct for disease misclassification in epidemiologic studies. *Am J Epidemiol*. 1993;138(11):1007–15.
92. Hutcheon JA, Chiolero A, Hanley JA. Random measurement error and regression dilution bias. *BMJ*. 2010;340:c2289.
93. Prospective Studies Collaboration. Blood cholesterol and vascular mortality by age, sex, and blood pressure: a meta-analysis of individual data from 61 prospective studies with 55 000 vascular deaths. *Lancet*. 2007;370(9602):1829–1839.
94. Prospective Studies Collaboration. Age-specific relevance of usual blood pressure to vascular mortality: a meta-analysis of individual data for one million adults in 61 prospective studies. *Lancet*. 2002;360(9349):1903–1913.
95. Hunter DJ, Spiegelman D, Adami HO, et al. Cohort studies of fat intake and the risk of breast cancer--a pooled analysis. *New Engl J Med*. 1996;334(6):356–61.
96. Rosner B, Spiegelman D, Willett WC. Correction of Logistic Regression Relative Risk Estimates and Confidence Intervals for Random Within-Person Measurement Error. *Am J Epidemiol*. 1992;136(11):1400–1413.
97. Carroll RJ, Stefanski LA. Measurement error, instrumental variables and corrections for attenuation with applications to meta-analyses. *Stat Med*. 1994;13(12):1265–82.
98. Kaaks R, Plummer M, Riboli E, Esteve J, van Staveren W. Adjustment for bias due to errors in exposure assessments in multicenter cohort studies on diet and cancer: a calibration approach. *Am J Clin Nutr*. 1994;59(1):245S–250.
99. Subar AF, Thompson FE, Kipnis V, et al. Comparative validation of the Block, Willett, and National Cancer Institute food frequency questionnaires : the Eating at America's Table Study. *Am J Epidemiol*. 2001;154(12):1089–99.
100. Yeung EH, Saudek CD, Jahren AH, et al. Evaluation of a novel isotope biomarker for dietary consumption of sweets. *Am J Epidemiol*. 2010;172(9):1045–52.
101. Männistö S, Smith-Warner SA, Spiegelman D, et al. Dietary carotenoids and risk of lung cancer in a pooled analysis of seven cohort studies. *Cancer Epidemiol Biomarkers Prev*. 2004;13(1):40–8.
102. Lee DH, Folsom AR, Jacobs Jr. DR. Dietary iron intake and Type 2 diabetes incidence in postmenopausal women: the Iowa Women's Health Study. *Diabetologia*. 2004;47(2):185–194.
103. Higgins JP, Thompson SG. Quantifying heterogeneity in a meta-analysis. *Stat Med*. 2002;21(11):1539–1558.
104. Newcombe RG. Two-sided confidence intervals for the single proportion: comparison of seven methods. *Stat Med*. 1998;17(8):857–72.
105. Eypasch E, Lefering R, Kum CK, Troidl H. Probability of adverse events that have not yet occurred: a statistical reminder. *BMJ*. 1995;311(7005):619–20.
106. Duval S, Tweedie R. A Nonparametric " Trim and Fill " Method of Accounting for Publication Bias in Meta-Analysis. *J Am Stat Assoc*. 2000;95(449):89–98.
107. Bleich SN, Wolfson JA, Vine S, Wang YC. Diet-beverage consumption and caloric intake among US adults, overall and by body weight. *Am J Public Heal*. 2014;104(3):e72–8.
108. National Center for Health Statistics N. National Health and Nutrition Examination Survey, NHANES (2009-2010). 2005(April 28, 2005). Available at: http://www.cdc.gov/nchs/nhanes.htm.
109. Public Health England and Food Standards Agency. National Diet and Nutrition Survey: results from Years 1 to 4 (combined) of the rolling programme for 2008 and 2009 to 2011 and 2012. 2014. Available at: https://www.gov.uk/government/publications/national-diet-and-nutrition-survey-results-from-years-1-to-4-combined-of-the-rolling-programme-for-2008-and-2009-to-2011-and-2012.
110. Greenland S. Estimation of Population Attributable Fractions from Fitted Incidence Ratios and Exposure Survey Data, with an Application to Electromagnetic Fields and Childhood Leukemia. *Biometrics*. 2001;57(1):182–188.
111. US Burden of Disease Collaborators. The state of us health, 1990-2010: Burden of diseases, injuries, and risk factors. *JAMA*. 2013;310(6):591–608.
112. Lim SS, Gaziano T a, Gakidou E, et al. Prevention of cardiovascular disease in high-risk individuals in low-income and middle-income countries: health effects and costs. *Lancet*. 2007;370(9604):2054–62.
113. Schünemann AHJ, Oxman AD, Vist GE, Higgins JPT, Jonathan J. Chapter 12 : Interpreting results and drawing conclusions. In: Higgins J, Green S, eds. *Cochrane Handbook for Systematic Reviews of Interventions*. West Sussex, England: The Cochrane Collaboration; 2008:1–24.
114. Selvin E, Parrinello CM, Sacks DB, Coresh J. Trends in Prevalence and Control of Diabetes in the United States, 1988–1994 and 1999–2010. *Ann Intern Med*. 2014;160(8):517.
115. Souverein OW, Dekkers AL, Geelen A, et al. Comparing four methods to estimate usual intake distributions. *Eur J Clin Nutr*. 2011;65 Suppl 1:S92–101.
116. Schmidt MI, Duncan BB, Bang H, et al. Identifying Individuals at High Risk for Diabetes: The Atherosclerosis Risk in Communities study. *Diabetes Care*. 2005;28(8):2013–2018.
117. Mann DM, Bertoni AG, Shimbo D, et al. Comparative validity of 3 diabetes mellitus risk prediction scoring models

in a multiethnic US cohort: the Multi-Ethnic Study of Atherosclerosis. *Am J Epidemiol*. 2010;171(9):980–988.
118. Heron M. Deaths: Leading Causes for 2010. *Nat Vital Stat Rep*. 2013;62(6):1–97.
119. United Kingdon Office for National Statistics. Revised Annual Mid-year Population Estimates, 2001 to 2010. 2013. Available at: http://www.ons.gov.uk/ons/rel/pop-estimate/population-estimates-for-uk--england-and-wales--scotland-and-northern-ireland/mid-2001-to-mid-2010-revised/stb---mid-2001-to-mid-2010-uk-revised-population-estimates.html#tab-Key-Points.
120. The QDiabetes® algorithm, version 2013.0. *ClinRisk Ltd*. 2013. Available at: http://www.qdscore.org/index.php.
121. Hippisley-Cox J, Coupland C, Robson J, Sheikh A, Brindle P. Predicting risk of type 2 diabetes in England and Wales: prospective derivation and validation of QDScore. *BMJ*. 2009;338:b880.
122. Collins GS, Altman DG. External validation of QDSCORE(®) for predicting the 10-year risk of developing Type 2 diabetes. *Diab Med*. 2011;28(5):599–607.
123. International Diabetes Federatoin. The Global Burden. In: *IDF Diabetes Atlas*. 6th ed. Brussels, Belgium: International Diabetes Federation; 2013:29–49.
124. Basu S, McKee M, Galea G, Stuckler D. Relationship of soft drink consumption to global overweight, obesity, and diabetes: a cross-national analysis of 75 countries. *Am J Public Heal*. 2013;103(11):2071–7.
125. Greenland S. Interval estimation by simulation as an alternative to and extension of confidence intervals. *Int J Epidemiol*. 2004;33(6):1389–97.
